# Genetic Code Expansion in *Shewanella oneidensis* MR-1 Allows Site-Specific Incorporation of Bioorthogonal Functional Groups into a *c*-Type Cytochrome

**DOI:** 10.1021/acssynbio.4c00248

**Published:** 2024-08-19

**Authors:** Colin W. J. Lockwood, Benjamin W. Nash, Simone E. Newton-Payne, Jessica H. van Wonderen, Keir P. S. Whiting, Abigail Connolly, Alexander L. Sutton-Cook, Archie Crook, Advait R. Aithal, Marcus J. Edwards, Thomas A. Clarke, Amit Sachdeva, Julea N. Butt

**Affiliations:** † School of Chemistry and School of Biological Sciences, University of East Anglia, Norwich Research Park, Norwich NR4 7TJ, U.K.; ‡ School of Life Sciences, University of Essex, Colchester CO4 3SQ, U.K.

**Keywords:** amber suppression, extracellular electron transfer, cytochrome, genetic code expansion, *Shewanella*

## Abstract

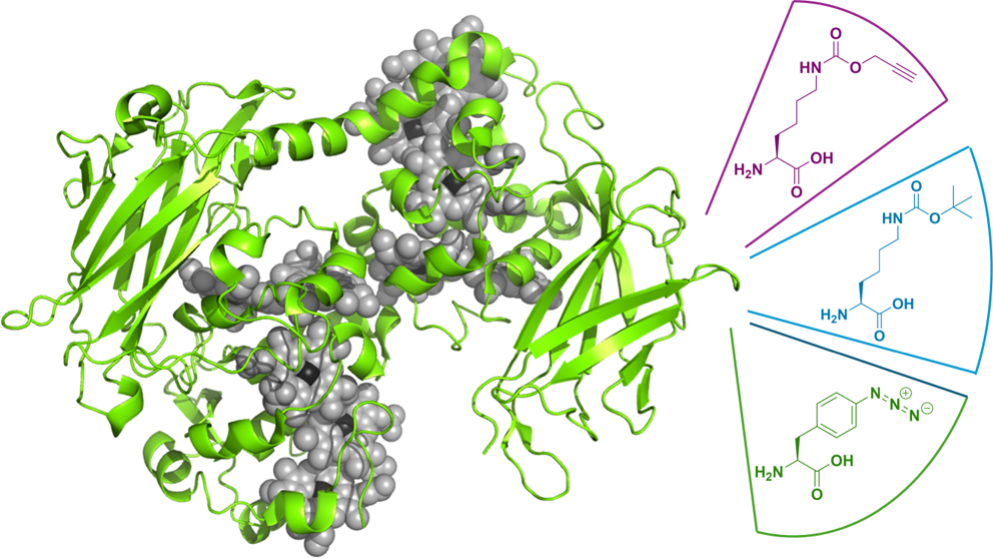

Genetic code expansion has enabled cellular synthesis of proteins containing unique chemical functional groups to allow the understanding and modulation of biological systems and engineer new biotechnology. Here, we report the development of efficient methods for site-specific incorporation of structurally diverse noncanonical amino acids (ncAAs) into proteins expressed in the electroactive bacterium *Shewanella oneidensis* MR-1. We demonstrate that the biosynthetic machinery for ncAA incorporation is compatible and orthogonal to the endogenous pathways of *S. oneidensis* MR-1 for protein synthesis, maturation of *c*-type cytochromes, and protein secretion. This allowed the efficient synthesis of a *c*-type cytochrome, MtrC, containing site-specifically incorporated ncAA in *S. oneidensis* MR-1 cells. We demonstrate that site-specific replacement of surface residues in MtrC with ncAAs does not influence its three-dimensional structure and redox properties. We also demonstrate that site-specifically incorporated bioorthogonal functional groups could be used for efficient site-selective labeling of MtrC with fluorophores. These synthetic biology developments pave the way to expand the chemical repertoire of designer proteins expressed in *S. oneidensis* MR-1.

## Introduction

Bacterial multiheme cytochromes (MHCs) attract much attention for their contributions to extracellular electron transfer whereby electrons are exchanged across the cell boundary and consequently between internal enzymes and external redox partners.^1−6^ These processes evolved to allow the capture of electrons for anabolic reactions and the release of electrons from internal oxidation during anaerobic respiration. For biotechnology, it is significant that the extracellular redox partner can be an electrode. As a consequence, MHCs and extracellular electron transfer contribute to electricity production by microbial fuel cells, support microbial electrosynthesis, allow biosensing, and enable living electronics.^7−11^ Opportunities to use purified MHCs as sustainable electronic components^11−13^ and in light-driven microreactors^14^ have also been noted. To further advance our understanding and deployment of MHCs, it is of interest to equip these proteins with novel properties, for example, by expanding their chemistry beyond that afforded by the 20 canonical amino acids. Such approaches would provide new prospects to probe, control, and redesign the function of MHCs. Indeed, more than 200 noncanonical amino acids (ncAAs) are now reported along with robust methods for their incorporation into proteins by genetic code expansion.^15−19^

Amber stop codon suppression methodology^18−23^ is the most frequently used approach to the genetic encoding of ncAAs. ncAA incorporation is achieved by providing the cells with an aminoacyl-tRNA synthetase/tRNA pair (aaRS/tRNA^cua^) that has the following properties: (1) the aaRS is specific to the ncAA, (2) the tRNA^cua^ binds to the amber (UAG) stop codon, and (3) the aaRS/tRNA pair is orthogonal to the host aaRS/tRNA pairs. This approach is routinely applied with *Escherichia coli* to introduce ncAAs into proteins that require noncovalently bound *b*-type heme to function, e.g., myoglobin,^24^ cytochromes P450,^25^ peroxidase,^26^ and ascorbate peroxidase.^27^ There are far fewer reports of incorporation of ncAAs into the family of proteins defined by the presence of *c*-type heme to which MHCs belong. This is partly due to the difficulty of forming covalent thioether linkages between the protoporphyrin cofactor and Cys residues in the canonical CxxCH *c*-type heme binding site. A dedicated cytochrome *c* maturation machinery is required for this attachment and typically overexpressed from a plasmid to accompany the production of *c*-type cytochromes in *E. coli* ^28,29^ A similar approach has been used for the few reported examples of ncAA incorporation into such proteins. Monoheme cytochromes *c* containing the ncAA *p*-cyanophenylalanine^30^ and *p*-carboxymethyl-l-phenylalanine^31^ were produced with coexpression of yeast heme lyase to facilitate covalent attachment of the heme. A tetraheme containing cytochrome *c*_3_ with the ncAA *para*-propargyloxyphenylalanine was produced with overexpression of the *E. coli* maturation proteins CcmA-H.^32^ In each of these scenarios, multiple plasmids imparting differing antibiotic resistance were used to accommodate genes for the protein of interest, the cytochrome maturation machinery, and the aaRS/tRNA^CUA^ pair.^30−32^

**Figure 1:**
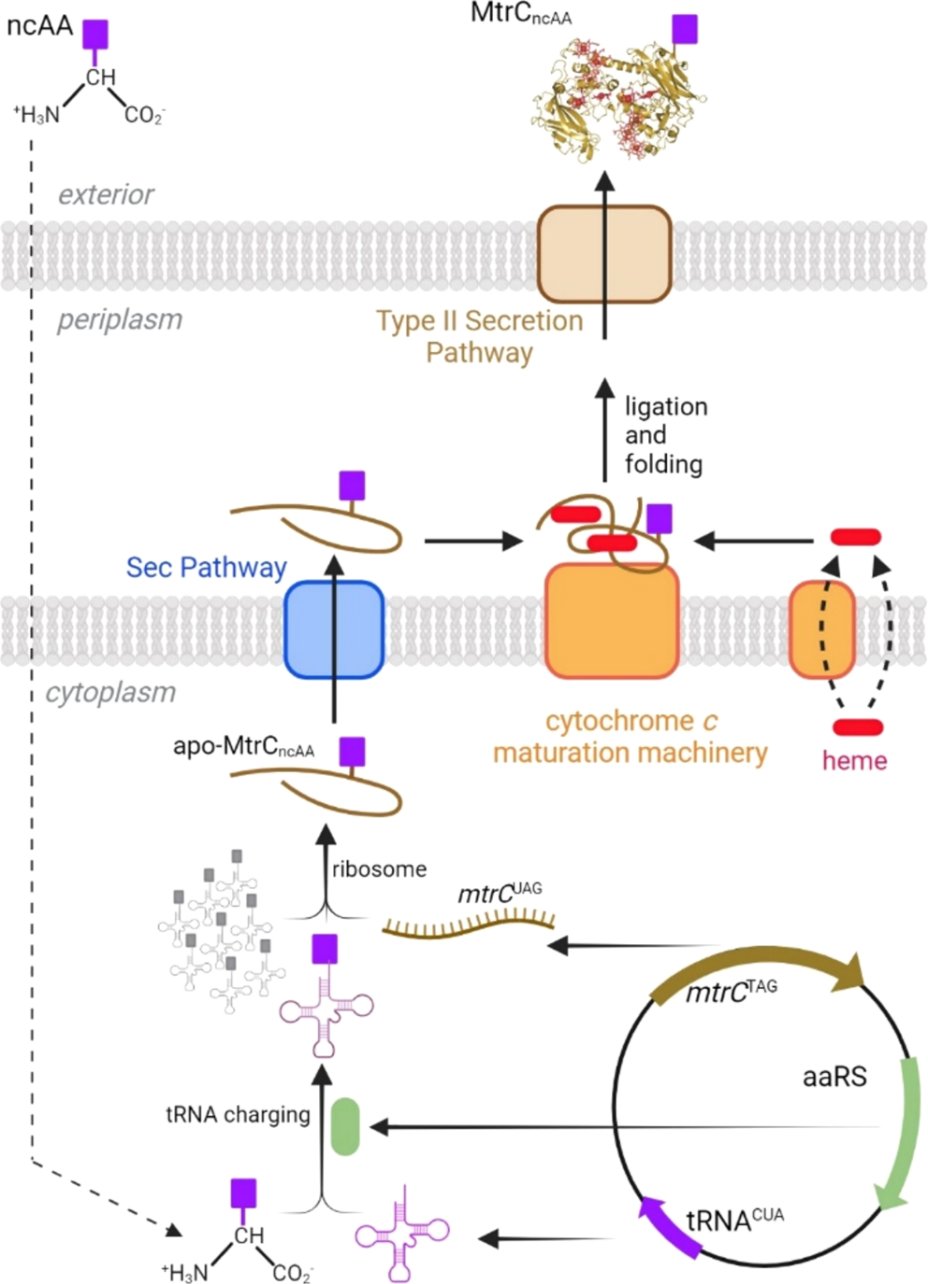
Schematic illustrating the production, maturation, and secretion of ncAA-containing MtrC protein by MR-1. A single plasmid encodes an amber stop codon variant of the *mtrC* gene and the orthogonal aaRS/tRNA pair (*Mb*PylRS/PylT or *Mj*CNFRS/tRNA), enabling directed ncAA insertion. Expression of the ncAA insertion system in the presence of exogenous ncAA generates apo-MtrC_ncAA_. The apo-MtrC_ncAA_ undergoes translocation to the periplasm via the Sec pathway (blue). Covalent attachment of heme prosthetic groups and folding by the cytochrome maturation machinery (orange) forms holo-MtrC_ncAA_. Holo-MtrC_ncAA_ is exported across the outer membrane by the Type II secretion system (brown) and released to the medium. Created with BioRender.com.

**Figure 2:**
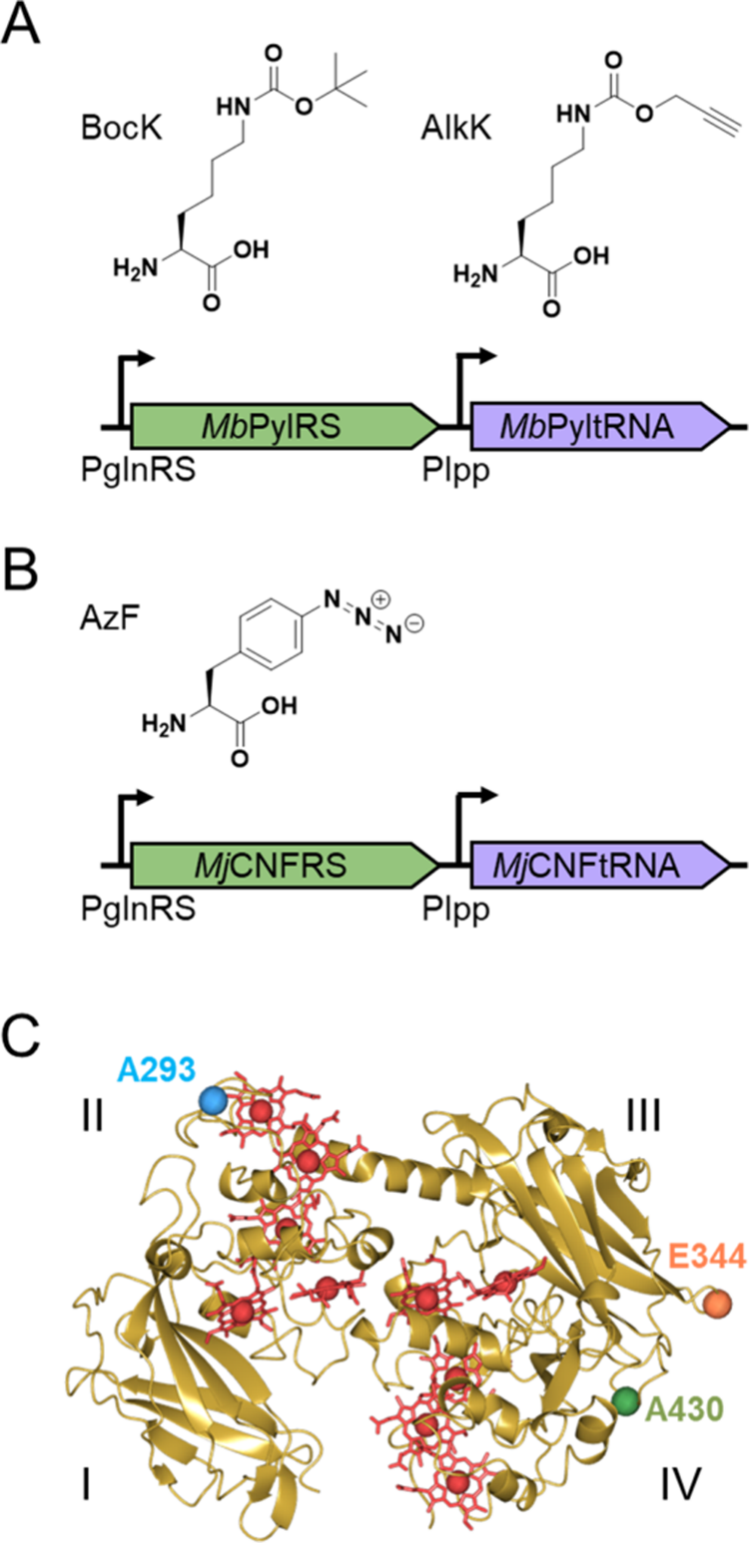
ncAA insertion systems used in our studies. (A) *Mb*PylRS/PyltRNA^CUA^ pair incorporates N^ε^-Boc-l-lysine (BocK) and N^ε^-(4-pentynyloxycabonyl)-l-lysine (AlkK). (B) *Mj*CNFRS/tRNA pair incorporates *p-*azido-l-phenylalanine (AzF). (C) Crystal structure of wtMtrC (PDB ID: 4LM8) indicating domains I–IV, the 10 covalently attached *c*-hemes (red), and the locations of residues selected for replacement by ncAA (A293, E344, A430).

A Gram-negative γ-proteobacteria with unrivaled capacity for homologous and heterologous production of MHCs is *Shewanella oneidensis* MR-1 (MR-1).^6,33−36^ There is no need to coexpress plasmid encoded cytochrome *c* maturation machinery because the endogenous machinery supports the production of numerous, abundant MHCs.^6^ Thus, we investigated whether amber suppression in MR-1 could produce ncAA-containing MHCs and, specifically, variants of the MtrC protein, which contains ten *c*-type hemes.^33,37^ Our approach employed a single plasmid carrying the genes encoding for MtrC and the desired aaRS/tRNA^CUA^ pair, as illustrated schematically in Figure 1. Here, we report that this approach allowed the production of MtrC proteins containing site-specifically incorporated N^ε^-Boc-l-lysine (BocK) and N^ε^-(4-pentynyloxycabonyl)-l-lysine (AlkK), Figure 2A, expressed using the *Methanosarcina barkeri* pyrrolysyl-tRNA synthetase/tRNA_Pyl_^CUA^ (*Mb*PylRS/tRNA^CUA^) pair. We demonstrate site-specific incorporation of the tyrosine analogue, *p*-azido-l-phenylalanine (AzF) Figure 2B, into MtrC with evolved mutants of the *Methanocaldococcus jannaschii* tyrosyl-tRNA synthetase (*Mj*CNFRS) /tRNA^CUA^ pair. We also show that site-specific incorporation of ncAAs into MtrC occurs without disrupting the three-dimensional structure and electron transfer properties of the protein.

Our results reveal that the endogenous MR-1 machinery for MHC maturation and secretion is capable of efficient production of ncAA-containing MHCs using amber suppression. This finding paves the way to a deeper understanding of electron transfer across the MR-1 cell envelope by enabling methods that exploit the bioorthogonal chemistry of ncAAs.

## Results and Discussion

### Developing a Vector Encoding for a *c*-Type Cytochrome and a tRNA Synthetase/tRNA Pair

The MR-1 pathways of heme biosynthesis, apocytochrome transfer across the cytoplasmic membrane by the Sec system, and periplasmic cytochrome *c* maturation are illustrated schematically in Figure 1. Several predicted proteins in these pathways have genes terminated by an amber stop codon, as shown in Table S1. Introducing an aaRS/tRNA^CUA^ pair into MR-1 and growing the bacteria with a ncAA could lead to translational readthrough of these endogenous genes, which would negatively impact the maturation and secretion of MHCs. Thus, our first goal was to establish whether a wild-type MHC would be produced by MR-1 equipped with the capacity for amber stop codon suppression. For the latter functionality, we chose to use wild-type and evolved mutants of the *Mb*PylRS/tRNA^CUA^ pair^16,31^ and *Mj*CNFRS/tRNA^CUA^ pair^16,30,32^ that have been successfully used for site-specific incorporation of several ncAAs into proteins expressed in *E. coli*. As a representative MHC produced by MR-1, we chose to focus on production of the MtrC protein.^33,37^

MtrC is found on the outer surface of MR-1 cells.^38,39^ The mature protein has N-terminal lipidation^40,41^ and ten *c*-type hemes that are key to MtrC fulfilling its central role in the transfer of electrons from bacterial metabolism to external acceptors.^37,42−44^ Previously, to produce the large amounts of MtrC needed to facilitate structure determination and biophysical analysis, a system was utilized, which allows for the overexpression of MtrC as a soluble, secreted form that carries a C-terminal Strep II tag.^33^ The engineered *mtrC* gene was cloned downstream of an arabinose inducible promoter in a pBAD202D/TOPO plasmid, Figure S1A, and the resulting plasmid is termed pBAD.C hereafter. Transformation of MR-1 with pBAD.C gives strain MR-1.C that produces soluble MtrC. That protein, termed wtMtrC hereafter, is purified with a yield of 5–10 mg per liter of the culture.^33^ To examine if the *Mb*PylRS/tRNA^CUA^^16,31^ and *Mj*CNFRS/tRNA^CUA^ pairs^16,30,32^ interfere with the maturation and transport of wtMtrC in MR-1, wtMtrC was expressed in the background of these aaRS/tRNA pairs and their corresponding ncAAs. DNA fragments^16^ corresponding to *Mb*PylRS/tRNA^CUA^, Figure 2A, or *Mj*CNFRS/tRNA^CUA^, Figure 2B, were inserted into pBAD.C. This resulted in two plasmids. pBAD.Pyl.C contained genes for wtMtrC and the *Mb*PylRS/tRNA^CUA^ pair, Figure S1B. pBAD.*Mj*.C contained genes for wtMtrC and *Mj*CNFRS/tRNA^CUA^ pair, Figure S1C. Plasmids and strains used in this study are summarized in Table S2.

**Figure 3:**
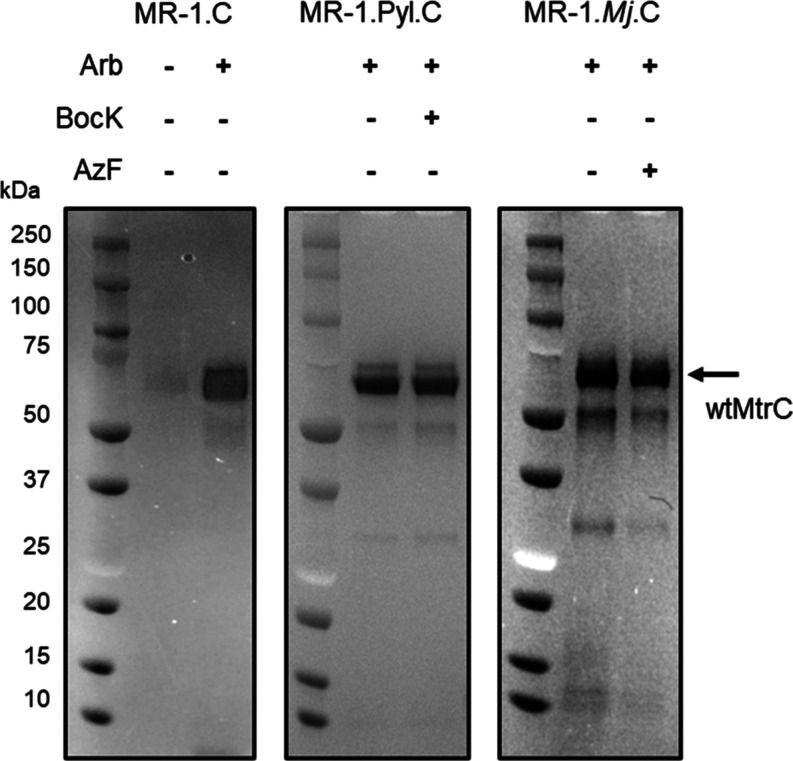
wtMtrC expression in the presence of ncAA insertion systems and ncAAs. SDS-PAGE gel images for spent medium from cultures of MR-1.C, MR-1.Pyl.C, and MR-1.*Mj*.C that were grown with and without arabinose (Arb), BocK, and AzF, as indicated. Proteins visualized by heme stain. The arrow labeled MtrC indicates the expected migration of wtMtrC protein. For each gel, the left lane contains MW markers. Gel images for the same samples resolved by SDS-PAGE with proteins visualized by Coomassie stain are presented in Figure S2.

Strain MR-1.Pyl.C (MR-1 carrying pBAD.Pyl.C) was cultured in the presence of arabinose with and without BocK. Sodium dodecyl-sulfate polyacrylamide gel electrophoresis (SDS-PAGE) analysis of the spent medium with *c*-type cytochromes visualized through peroxidase-linked heme stain, Figure 3 center, revealed bands having the same migration as wtMtrC. Affinity chromatography, followed by liquid chromatography–mass spectrometry (LC–MS) of the purified proteins, Table 1, confirmed the presence of wtMtrC. Similar results were obtained with strain MR-1.*Mj*.C (MR-1 carrying pBAD.*Mj*.C) cultured with arabinose in the presence and absence of AzF, Figure 3 right, Table 1. For each strain and culture condition, the yield of wtMtrC was 5–10 mg per liter of the culture, Table 1. Thus, there was no evidence that our selected aaRS/tRNA^CUA^ pairs and ncAAs impacted the maturation and secretion of MtrC through translational readthrough of endogenous genes.

**Table 1:** Yields and Intact Mass Values for Purified MtrC Proteins

MR-1 strain	ncAA in culture media	yield of MtrC protein (mg/L)	observed intact mass (Da)	predicted intact mass^a^ (Da)	protein^a^
Pyl.C	BocK	10	76,256	76,252	wtMtrC
Pyl.C_293UAG_	BocK	0.8	76,412	76,409	MtrC_293BocK_
Pyl.C_344UAG_	BocK	0.8	76,354	76,351	MtrC_344BocK_
Pyl.C_430UAG_	BocK	0.9	76,412	76,409	MtrC_430BocK_
Pyl.C_344UAG_	AlkK	0.4	76,337	76,334	MtrC_344AlkK_
*Mj*.C	AzF	6.1	76,255	76,252	wtMtrC
*Mj*.C_293UAG_	AzF	5.0	76,346	76,343	MtrC_293AmF_
			76,371	76,369	MtrC_293AzF_
*Mj*.C_344UAG_	AzF	1.8	76,314	76,311	MtrC_344AzF_
*Mj*.C_430UAG_	AzF	2.5	76,372	76,369	MtrC_430AzF_
*Mj*.C_293UAG_	none	8.6	76,332	76,328	MtrC_293Phe_
*Mj*.C_344UAG_	none	1.0	76,278	76,270	MtrC_344Phe_
*Mj*.C_430UAG_	none	1.9	76,334	76,328	MtrC_430Phe_

a MtrC proteins and their intact masses were predicted from the strain and culture condition. When the observed mass of the purified protein differed significantly from that prediction, the observed mass, together with the strain and culture condition, was used to identify the MtrC protein.

To compare the general fitness of the strains carrying the aaRS/tRNA^CUA^ pairs, the growth of MR-1.Pyl.C and MR-1.*Mj*.C was monitored by measuring the optical density of cultures at 600 nm, Figure S3. While the growth of these strains in the early exponential phase is comparable to that of MR-1.C, addition of ncAAs seems to have a negative impact on growth, suggesting that the charged tRNA has some toxicity. This might be due to the undesired incorporation of ncAAs into endogenous proteins in MR-1. Recoding MR-1 to replace TAG stop codons in endogenous genes with synonymous codons could potentially reduce the toxicity. However, these investigations are beyond the scope of the present study. Importantly, despite some toxicity, significant amounts of cells are obtained to assess the utility of PyRS/tRNA^CUA^ and *Mj*RS/tRNA^CUA^ pairs in site-specific incorporation of ncAAs into proteins expressed in MR-1.

### Production of MtrC Containing BocK and AzF

To establish that the *Mb*PylRS/tRNA^CUA^ and *Mj*CNFRS/tRNA^CUA^ pairs were functional in MR-1, we aimed to introduce ncAAs into wtMtrC. The structure of wtMtrC was inspected to identify surface residues that might be changed to ncAAs with limited effect on the MtrC structure. Residue A293 in Domain II and E344 and A430 in Domain III were chosen, Figure 2C. Native codons corresponding to those sites were mutated to the amber stop codon (TAG) in pBAD.Pyl.C, resulting in plasmids termed pBAD.Pyl.C_xxxUAG_, where xxx specifies position 293, 344, or 430 in wtMtrC. Transformation of the plasmids into MR-1 produced strains MR-1. Pyl.C_xxxUAG_. A similar strategy produced strains MR-1.*Mj*.C_XXXUAG_ carrying the amber stop codon in pBAD.Mj.C. Mutagenic primers are summarized in Table S3.

**Figure 4:**
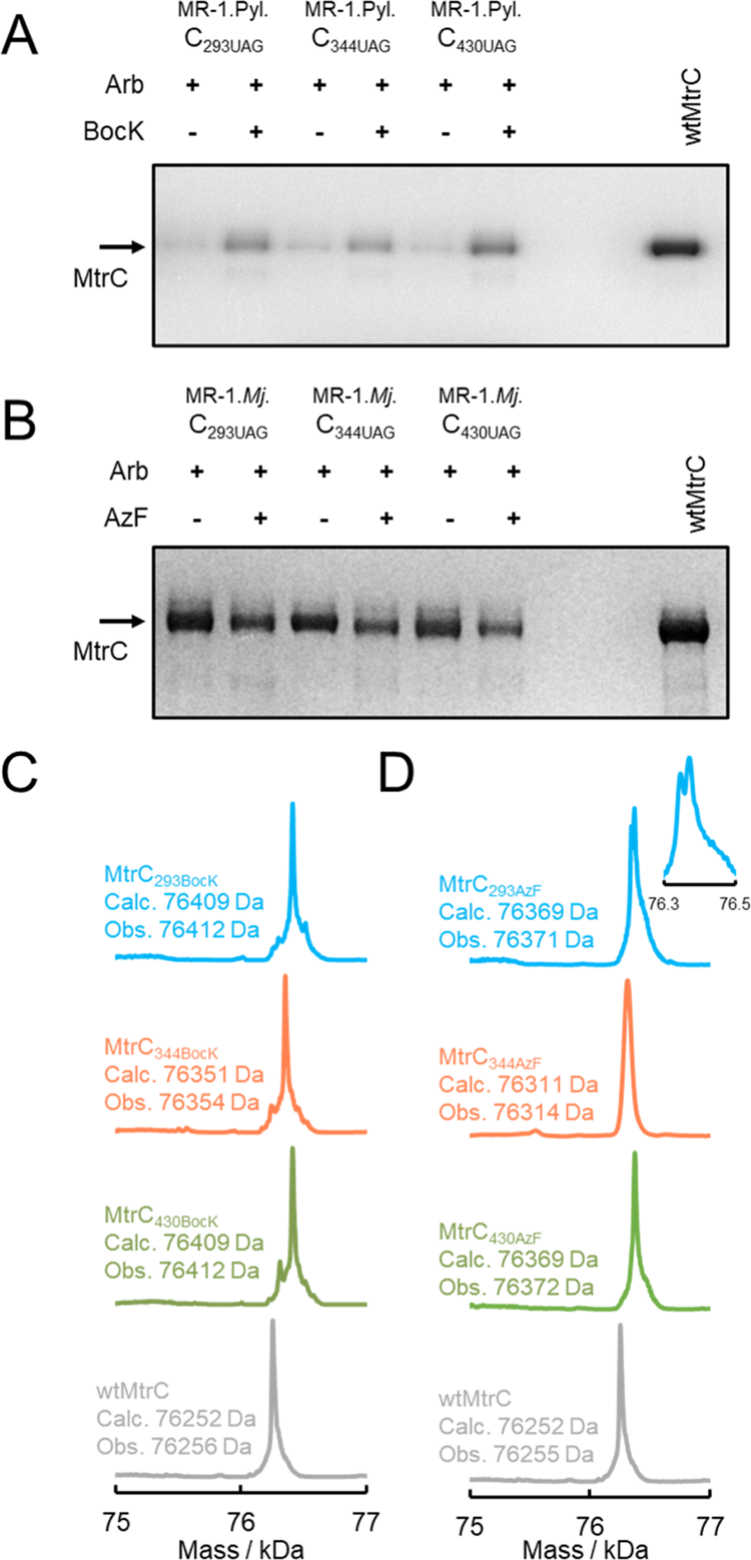
Production and characterization of ncAA-containing MtrC proteins. SDS-PAGE gel image with proteins visualized by heme stain for spent medium from (A) MR-1.PylC_xxxUAG_ strains cultured with and without arabinose and BocK, as indicated, and (B) MR-1.*Mj*.C_xxxUAG_ strains cultured with and without arabinose (Arb) and AzF, as indicated. The right lane contains wtMtrC purified from cultures of MR-1.C. Deconvoluted mass spectra for MtrC proteins purified from the spent medium of (C) MR-1.PylC_xxxUAG_ strains cultured with arabinose and BocK and (D) MR-1.*Mj*.C_xxxUAG_ strains cultured with arabinose and AzF. Spectra are labeled with details of the predicted ncAA-containing protein, and the spectrum (gray) of wtMtrC is included for reference. Calculated (calc.) and observed (obs.) intact mass values are included. For panels (A) and (B), gel images for samples with the proteins visualized by Coomassie stain are presented in Figure S4.

Using MR-1.Pyl.C amber mutant strains, protein expression was induced with arabinose in the absence or presence of BocK. After overnight culture, SDS-PAGE analysis of the spent medium, Figure 4A, revealed significantly more intense bands for MtrC in the presence of BocK. The identity of purified MtrC_293BocK_, MtrC_344BocK_, and MtrC_430BocK_ from the corresponding MR-1.Pyl.C_xxxUAG_ strain was confirmed by LC–MS, Figure 4C and Table 1. Taken together, SDS-PAGE and LC–MS analysis demonstrate the site-specific incorporation of ncAA and BocK, at three distinct positions in MtrC.

In contrast to the PylRS/tRNA pair, when amber suppression was performed using the *Mj*CNFRS/ *Mj*tRNA^CUA^ pair, similar levels of full-length MtrC were observed in the presence and absence of AzF, Figure 4B. As a consequence, Strep II-tagged proteins were recovered from all cultures and analyzed by LC–MS. First, we consider the properties of proteins from the culture in the presence of AzF. *Mj*.C_344AzF_ and *Mj*.C_430AzF_ were produced as essentially homogeneous samples, Figure 4D, with intact mass values indicative of AzF incorporation, Table 1. In contrast, the deconvoluted mass spectrum of the protein recovered from MR-1.*Mj*.C_293UAG_ revealed two components, Figure 4D. The intact mass, Table 1, for one component was in good agreement with the predicted molecular weight of the azide containing protein, *Mj*.C_293AzF_. The second component was 25 Da lighter, as shown in Table 1. Similar behavior has been observed when incorporating AzF in other proteins^45,46^ and attributed to the presence of *p*-amino-l-phenylalanine (AmF) formed on reduction of AzF. Thus, we assign the lighter MtrC protein to be *Mj*.C_293AmF_. In considering the stability of AzF, it may be significant that residue 293 lies approximately 13 Å from the Heme 5 porphyrin ring, whereas residues 344 and 430 are more than 22 Å from the closest heme, Figure 2C. Reduction of AzF as residue 293 may then be facilitated by Heme 5 redox cycling. However, further investigation of this behavior was beyond the scope of this study.

MtrC proteins recovered from the culture in the absence of AzF were homogeneous, as shown in Figure S5, and had the masses expected for Phe incorporated at the residue encoded by the amber codon, Table 1. The most reasonable interpretation is that *Mj*tRNA^cua^ is charged by Phe using *Mj*CNFRS or endogenous Phe-aaRS in MR-1 in the absence of ncAA AzF. Similar behavior has been reported in other studies.^47^ Importantly for the production of ncAA-containing MtrC proteins and as described above, the culture, in the presence of 4 mM AzF, ensures the aaRS/tRNA^CUA^ pair achieves preferential insertion of the desired ncAA into MtrC.

### Site-Specific Replacement of MtrC Surface Residues with BocK Does Not Disrupt the Structure or Electron Transfer Properties

**Figure 5:**
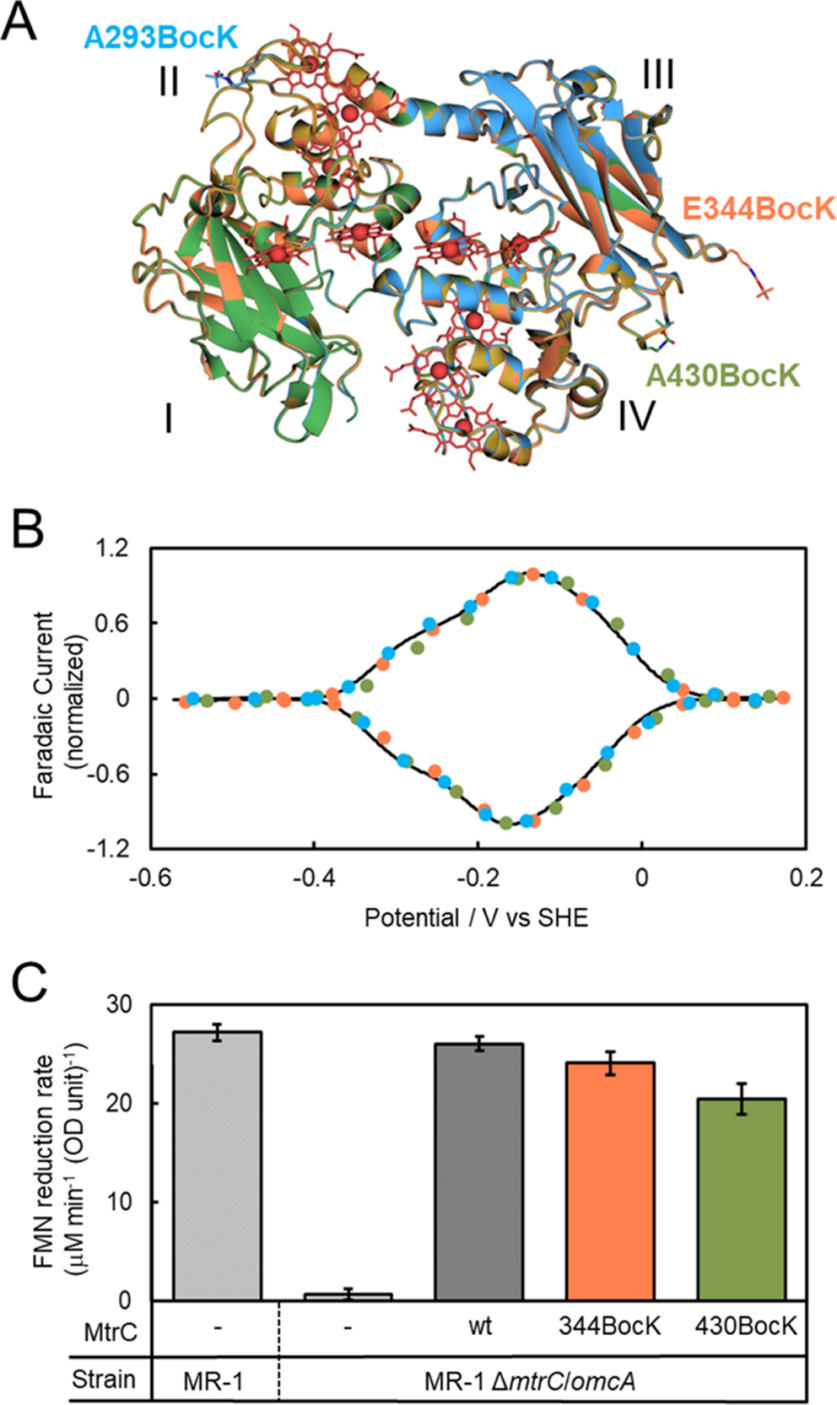
Structures and biophysical characterization of BocK-containing MtrC_BocK_ proteins. (A) Pairwise alignment of the crystal structures of MtrC_293BocK_ (blue), MtrC_344BocK_ (coral), and MtrC_430BocK_ (green) with wtMtrC (gold) using Superpose. For clarity, only the wtMtrC hemes are displayed. Protein domains I–IV are identified. (B) Faradic currents from protein film cyclic voltammetry of wtMtrC (black line), MtrC_293BocK_ (blue circles), MtrC_344BocK_ (coral circles), and MtrC_430BocK_ (green circles). Scan rate 30 mV s^–1^. Buffer-electrolyte 50 mM 4-(2-hydroxyethyl)-1-piperazineethanesulfonic acid (HEPES), 100 mM NaCl, pH 7.0. (C) Rates of flavin mononucleotide (FMN) reduction for the indicated MR-1 strains cultured with and without wtMtrC, MtrC_344BocK_, and MtrC_430BocK_, as indicated. Error bars represent standard error from three independent replicates.

Structures for the three BocK-containing MtrC proteins described above were resolved by X-ray crystallography. Diffracting crystals were obtained under conditions similar to those used to solve the structure of wtMtrC.^37^ The structures were resolved to 2.00, 1.90, and 1.81 Å for BocK as residues 293 (PDB ID: 8QC9), 344 (PDB ID: 8QBZ), and 430 (PDB ID: 8QBQ), respectively, Table S4. Superposition of the structures of the BocK-containing proteins and wtMtrC revealed no significant differences between the proteins, as shown in Figure 5A. A total main chain rmsd of ∼0.3 Å (MtrC_293BocK_ 0.24 Å, MtrC_344BocK_ 0.29 Å, and MtrC_430BocK_ 0.29 Å) was calculated using SUPERPOSE,^48^ and the positions of all ten heme cofactors overlay those in wtMtrC, Figure S6. Thus, site-specific replacement of MtrC surface residues with BocK has no discernible impact on the protein structure.

Redox properties of BocK-containing proteins were assessed by two approaches. For protein film electrochemistry, the purified proteins were adsorbed on hierarchical indium tin oxide electrodes and studied by cyclic voltammetry, Figure 5B. Reversible redox activity between approximately 0.1 and −0.4 V was revealed. The current–potential profiles of the peaks for reduction (negative current) and oxidation (positive current) for all three BocK-containing proteins were highly similar to those of wtMtrC.

To assess protein redox activity in a cellular context, the ability of MtrC variants to restore extracellular reduction of flavin mononucleotide (FMN) to an MR-1 deletion strain was investigated, Figure 5C. Previously, Coursolle et al.^43,44^ reported that the ability of MR-1 to couple intracellular lactate oxidation with FMN reduction was significantly diminished by deletion of the genes for the extracellular MtrC and homologous OmcA cytochrome. We found that washed cells recovered from the culture of an Δ*mtrC/omcA* MR-1 strain^49^ augmented with wtMtrC displayed FMN reduction rates comparable to those of MR-1, Figure 5C. By contrast, there was no detectable FMN reduction for Δ*mtrC/omcA* MR-1 cells cultured in the absence of wtMtrC. Cells from cultures augmented by MtrC_344BocK_ and MtrC_430BocK_ also displayed FMN reduction rates comparable to those of MR-1, Figure 5C. Thus, the MtrC_344BocK_ and MtrC_430BocK_ proteins behave as wtMtrC. The most reasonable interpretation of the results is that all three proteins bind tightly to the external surface of Δ*mtrC/omcA* MR-1 cells to restore the pathway for electron transfer from internal lactate oxidation to external FMN reduction. Thus, our results indicate that MtrC_344BocK_ and MtrC_430BocK_ retain the structure and redox activity of wtMtrC.

We conclude from the protein film electrochemistry and cell-based studies that incorporation of ncAAs onto the surface of MtrC occurs with a negligible impact on the structure and redox properties of that protein. MR-1 cells typically present MtrC tightly bound to the external face of the outer membrane spanning the MtrAB porin-cytochrome complex. A crystal structure of the homologous MtrCAB complex purified from *Shewanella baltica* OS185^42^ shows that MtrC Domain III, which contains residues 344 and 430, makes no contribution to the binding with MtrAB. This is consistent with the ability of wtMtrC, MtrC_344BocK_, and MtrC_430BocK_ to restore FMN reduction activity to Δ*mtrC/omcA* MR-1 cells by associating with MtrAB in the outer membrane. MtrC Domain II residues and, specifically, those near Heme 5 are expected to be intimately involved in the interface with MtrAB, as shown in Figure S7. For this reason, studies with the MtrC_293BocK_ protein and the deletion strain were beyond the scope of this study.

### Site-Specific Labeling of MtrC Surface ncAAs with Fluorescent Probes Using Bioorthogonal Reactions

**Figure 6:**
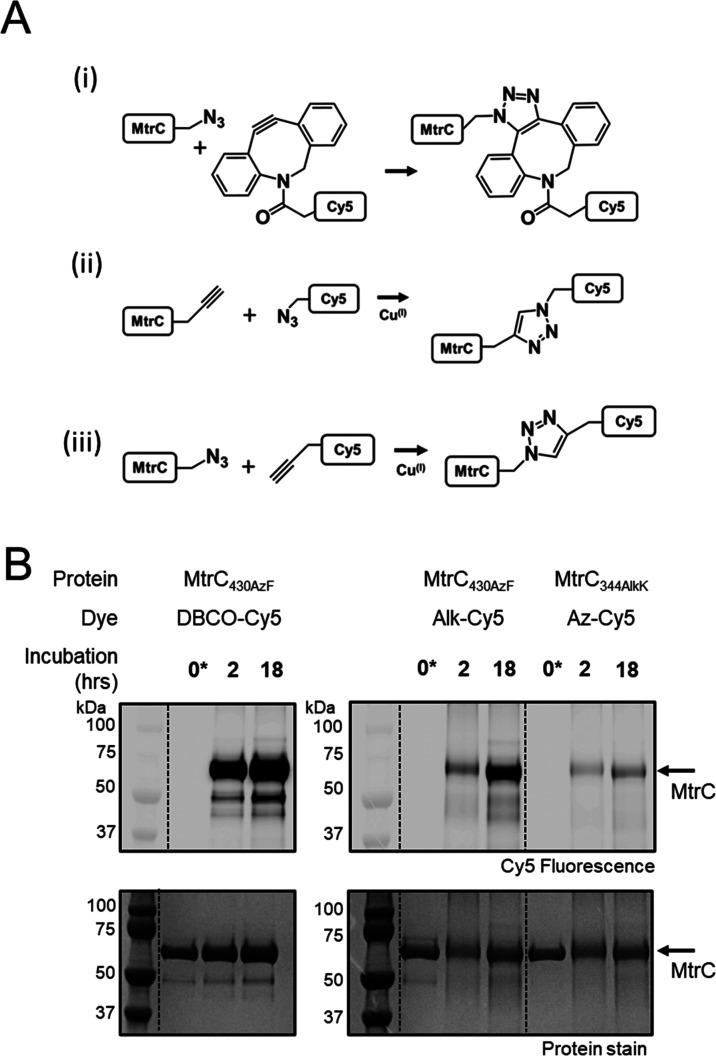
Introduction of fluorescent probes to ncAA-containing MtrC proteins using bioorthogonal chemistry. (A) Reactions explored in this study. (i) Strain-promoted azide–alkyne cycloaddition with dibenzocyclooctyne sulfo-cyanine 5 (DBCO-Cy5). (ii), (iii) Copper-catalyzed azide–alkyne cycloaddition with functionalized Cy5 dyes. (B) SDS-PAGE gel images for samples of ncAA-containing MtrC proteins incubated with functionalized Cy5 dyes, as indicated. Upper panel: Cy5 dye visualized by fluorescence emission (excitation at 635 nm). Lower panel: proteins visualized by Coomassie stain. Arrows indicate the expected migration of MtrC proteins.

Experiments to assess whether ncAAs on the surface of MtrC could undergo bioorthogonal reactions were performed with functionalized forms of the fluorescent probe sulfo-cyanine 5 (Cy5). The spontaneous strain-promoted azide–alkyne click reaction, Figure 6A-i, was investigated with dibenzocyclooctyne functionalized Cy5 dye. SDS-PAGE analysis of the reaction products with the gels visualized by Cy5 fluorescence revealed bands at the expected location for Cy5-labeled MtrC_430AzF_, Figure 6B left. Equivalent experiments confirmed labeling of the MtrC_293AzF_ and MtrC_344AzF_ proteins, Figure S8. An alkyne functionalized Cy5 dye was used to investigate Cu(I) catalyzed azide–alkyne cycloaddition in the presence of ascorbate and tris(3-hydroxypropyl triazolylmethyl)amine, Figure 6A-ii. The formation of a MtrC_430AzF_-Cy5 conjugate was again confirmed by SDS-PAGE analysis of the reaction products, Figure 6B center.

We also prepared MtrC_344AlkK_ for a further test of the ability of ncAA-containing MtrC proteins to undergo bioorthogonal reactions, Figure 6A-iii. LC–MS confirmed the production of this alkyne containing protein from a culture of MR-1.Pyl.C_344UAG_ in the presence of AlkK, Table 1. Formation of Cy5-labeled protein occurred on incubation of MtrC_344AlkK_ and azido-functionalized Cy5 dye, Figure 6B right. Control experiments with BocK-containing MtrC proteins failed to produce evidence of labeling with any of the Cy5 dyes used here, e.g., Figure S9. Thus, MtrC proteins containing surface AzF and AlkK can be site-selectively labeled with Cy5 dyes through bioorthogonal reactions.

Electronic absorbance spectra of the oxidized, i.e., air-equilibrated, AzF- and AlkK-containing MtrC proteins present a prominent Soret band with maximum absorbance at 410 nm, Figure S10. A lower intensity Q-band is observed between 500 and 600 nm. When the spectra are normalized at 410 nm, they are indistinguishable from those of the crystallographically defined wtMtrC and BocK-containing MtrC proteins, Figure S10. The Soret- and Q-bands are sensitive to differences in the local environment and the ligation of heme cofactors since they arise from electronic transitions within those cofactors. Thus, the electronic absorbance spectra indicate that the AzF- and AlkK-containing proteins retain the 2° and 3° structure of wtMtrC and, consequently, we did not resolve the structures of these variant proteins by X-ray crystallography.

## Conclusions

We have expanded the genetic code of the electrogenic bacterium *S. oneidensis* MR-1. Using the *Mb*PylRS/tRNA^CUA^ and *Mj*CNFRS/tRNA^CUA^ pairs, we have incorporated both lysine and tyrosine ncAA analogues. We have deployed this system to site-specifically incorporate three different ncAAs at multiple locations into MtrC as a representative MHC that is additionally secreted from MR-1 cells. Site-specific replacement of surface residues with the ncAA BocK has minimal impact on the structural, redox, and functional properties of MtrC. Furthermore, using the expanded genetic code of MR-1, bioorthogonal functional groups were incorporated and exploited to site-specifically install fluorophores in MtrC. Given the utility of MR-1 for homologous and heterologous production of *c*-type cytochromes, our findings pave the way to using MR-1, in place of *E. coli*, for the production of ncAA-containing *c*-type cytochromes equipped with functionality not found in nature’s genetic alphabet.

## Methods

### General Reagents and Methods

Routine culturing of MR-1 strains was carried out in an LB medium (Formedium). For the production of MtrC, MR-1 strains were grown in M72 medium consisting of 5 g L^–1^ peptone from soybean meal (Merck), 15 g L^–1^ peptone from casein (Merck), and 5 g L^–1^ NaCl supplemented with 20 mM sodium lactate, 30 mM sodium fumarate, 25 mM HEPES, pH 7.8 and, where appropriate, kanamycin (30 μg mL^–1^). p-Azido-l-Phenylalanine (AzF) and (BocK) were obtained from Fluorochem Ltd. Alkyne lysine was synthesized in-house, following a previously described procedure.^45,50^ Sulfo-Cyanine 5 dyes (azide, alkyne, and dibenzocyclooctyne derivatives) were purchased from Antibodies.com and prepared as stock solutions (0.65–1 mM) in distilled water. SDS-PAGE used mPAGE 4–20% Bis-Tris Precast gels (Merck), and proteins were visualized by ReadyBlue Coomassie stain (Merck) or heme-dependent peroxidase activity.^51^ In-gel fluorescence from Cyanine 5 dyes was assessed with a Typhoon 9500 (GE Healthcare) imager with an excitation at 635 nm.

### Construction of pBAD.Pyl.C and pBAD.*Mj*.C Expression Plasmids

Plasmids and primers used in this study are listed in Tables S2 and S3, respectively. *Mb*PylRS/tRNA^CUA^ and *Mj*CNFRS/tRNA^CUA^ pairs were introduced into pBAD.C to create the amber suppression plasmids pBAD.Pyl.C and pBAD.*Mj*.C, respectively. pBAD.C, as previously described,^33^ is a pBAD202/D-TOPO-derived plasmid that contains the *mtrC* gene modified to encode for the signal peptide of MtrB from MR-1 and a C-terminal Strep II tag to aid purification. pBAD.C includes a basis of mobility (BOM) region containing a *Nde*1 restriction site into which the aaRS/tRNA^CUA^ pair was inserted. The BOM region facilitates transformation via conjugation, which could be sacrificed since it is not necessary for plasmid insertion into *E. coli* TOP10 or MR-1.

Plasmid pBAD.C was linearized by restriction digest at 37 °C using *Nde1* (New England Biolabs) and resolved on a 1% agarose gel. The band corresponding to the linearized backbone was excised, and DNA was isolated using a GenElute Gel Extraction Kit (Merck). DNA fragments containing the desired aaRS (on a gln *R*S promoter) and tRNA^CUA^ (on an lpp promoter) were amplified from AS61 and AS76 plasmids^16^ using primers that added flanking regions complementary to either side of the *NdeI* cut site within the pBAD.C plasmid. The amplified product containing the desired aaRS/tRNA^CUA^ pair was inserted into linearized pBAD.C using Gibson cloning, following the provided protocol (Gibson Assembly Cloning Kit, New England Biolabs). The resulting products were introduced to chemically competent *E. coli* Top10 cells with transformants isolated on LB Agar plates containing kanamycin at a concentration of 30 μg mL^–1^. Plasmids pBAD.Pyl.C and pBAD.*Mj*.C were purified from the transformants, and the presence of the desired aaRS/tRNA^CUA^ pair was confirmed by Sanger DNA sequencing (Eurofins). Plasmids were transformed by electroporation into MR-1 to create the kanamycin-resistant strains MR-1.Pyl.C and MR-1.*Mj*.C.

The amber stop codon was introduced into the *mtrC* gene of pBAD.Pyl.C and pBAD.*Mj*.C plasmids by PCR (Phusion Flash High-Fidelity PCR master Mix, Thermo Fisher Scientific) using the appropriate primers, Table S3. The resulting plasmids pBAD.Pyl.C_XXXUAG_ and pBAD.*Mj*.C_XXXUAG_ were propagated, sequenced, and introduced into MR-1, as described above.

### Purification of Strep II-Tagged MtrC Proteins

For routine production of MtrC proteins, single colonies of MR-1 containing the appropriate expression plasmid were used to inoculate 10 mL LB with kanamycin (30 μg mL^–1^) and grown aerobically overnight at 30 °C. These cultures provided the inoculum for 100 mL M72 medium supplemented with sodium lactate (20 mM), sodium fumarate (30 mM), and HEPES (25 mM) at pH 7.8 with kanamycin (30 μg mL^–1^).^33^ Cultures were grown aerobically with shaking at 180 rpm at 30 °C until an OD of 0.4 was reached (∼3 h). Expression of the *mtrC* gene was induced by the addition of arabinose to a final concentration of 5 mM. An appropriate volume of ncAA, 400 mM (at 100× concentration) in 1 M NaOH, was added to the culture to give a final concentration of 4 mM ncAA. The medium was then neutralized with 1 M HCl. Cultures were grown overnight at 30 °C, with shaking at 180 rpm. Spent medium containing the secreted MtrC was separated from cells by centrifugation (5000*g*, 4 °C, 20 min), and the supernatant was retained.

For each 100 mL of MtrC containing supernatant, 10 mL of 1 M Tris-HCl, 1.5 M NaCl, pH 8 was added, and the resulting solution was concentrated approximately 25 times using a 30 kDa MWCO cutoff spin concentrator (Merck). Concentrated medium was applied to a 1 mL Strep-Tactin Superflow FPLC column (IBA Lifesciences) pre-equilibrated with 100 mM Tris, 150 mM NaCl, pH 8 (Buffer A). After washing with 10 column volumes of Buffer A, bound proteins were eluted with 5 column volumes of 50 mM Biotin in Buffer A. The flow rate was 1 mL min^–1^ except when loading the column, and the flow rate was 0.5 mL min^–1^ when eluting the protein. Purified protein was exchanged into Buffer A using 30 kDa MWCO spin concentrators. Protein concentrations were determined by electronic absorbance spectroscopy using the Beer–Lambert law and an extinction coefficient of 1 260 mM^–1^ cm^–1^ for the air-equilibrated (oxidized) protein.^33^ Proteins were then snap frozen in liquid nitrogen and stored at −80 °C.

To produce BocK-containing MtrC proteins for crystallization and biophysical characterization, the above method was scaled to culture cells in 1 L of medium contained in a 2 L baffled flask. For each liter of clarified spent medium, 100 mL of 1 M Tris, 1.5 M NaCl, pH 8 was added, and the resulting solution was concentrated using a 30 kDa Vivaflow flow cassette concentrator (Sartorius). The concentrated medium was applied to a 5 mL Strep-Tactin Superflow FPLC column (IBA), and the column was developed as above. The flow rate was 5 mL min^–1^ unless loading the column or eluting protein when the flow rate was 1 mL min^–1^. Prior to crystallization, the affinity purified protein underwent gel filtration (1 mL min^–1^) using a HiLoad 16/60 Superdex 200 prep grade column, equilibrated with 20 mM HEPES, pH 7.8.

### LC–MS

Intact mass values were determined using a previously reported protocol.^41,52^ Samples containing ∼30 μM MtrC were diluted to 3 μM with aqueous acetonitrile (2% v/v) and formic acid (0.1% v/v) and loaded on a ProSwift RP-1S column (4.6 × 50 mm, Thermo Scientific) on an Ultimate 3000 uHPLC system (Dionex, Leeds, U.K.). The column was developed over 15 min with a linear gradient of acetonitrile (2% to 100%, v/v) in the presence of formic acid (0.1%, v/v). The column eluent was continuously introduced to a Bruker microQTOF-QIII mass spectrometer, controlled by Hyster (Bruker Daltonics), with positive electrospray ionization (ESI) and calibrated with an ESI-L tuning mix (Agilent Technologies). Data was analyzed using Compass Data Analysis, with Maximum Entropy v1.3, (Bruker Daltonics).

### Crystallographic Analysis of BocK-Containing MtrC Proteins

MtrC_344BocK_ and MtrC_430BocK_ were crystallized using conditions previously used to crystallize wtMtrC.^37^ Crystals were prepared by sitting-drop vapor diffusion with a reservoir solution of 0.2 M sodium acetate, 0.1 M CaCl_2_, pH 5.0 and 21% PEG 6000 and a protein concentration of 180 μM in 20 mM HEPES, pH 7.8. The drop volume was 0.6 μL formed by 1:1 and 2:1 (reservoir/protein) and incubated at 4 °C. MtrC_293BocK_ did not form crystals under the above conditions; therefore, a seeding strategy was adopted in which wtMtrC crystals grown under the above conditions were crushed using a pipette tip to form microcrystals. A seed stock was prepared by resuspending the microcrystals in the above crystallization solution. Drops were dispensed with a total volume of 0.6 μL formed of 5:6:1 reservoir/protein/seed stock. The seeding strategy produced crystals similar to those seen in the unseeded crystallization. Crystals were transferred into 0.2 M sodium acetate, 0.1 M CaCl_2_, pH 5.0, 21% PEG 6000, and 20% ethylene glycol to cryoprotect before being vitrified by plunging into liquid nitrogen.

Data were collected on MtrC crystals in a gaseous stream of nitrogen at 100 K on beamlines I24 and I04 at the Diamond Light Source (UK). MtrC crystals were of space group *P*2_1_2_1_2_1_ with typical cell dimensions of a = 52.97 b = 89.66 c = 153.55 Å. Data were processed using Xia2^53^ and were phased by molecular replacement in Phaser,^54^ using the wtMtrC structure as the search template (PDB ID: 4LM8). Coordinates have been deposited in the RCSB Protein Data Bank under accession codes 8QC9 (MtrC_293BocK_), 8QBZ (MtrC_344BocK_), and 8QBQ(MtrC_430BocK_).

### Protein Film Voltammetry

Experiments were carried out in an N_2_-filled chamber (atmospheric O_2_ < 5 ppm) using a three-electrode cell configuration inside a Faraday cage. The reference was an Ag/AgCl (saturated KCl) electrode, and measured potentials were corrected to value versus SHE by the addition of +197 mV. The counter electrode was provided by a length of Pt wire. The working electrode was hierarchical mesoporous indium tin oxide (ITO) prepared as described previously.^55^ A 10 μL aliquot of MtrC protein (approximately 40 μM in 50 mM MES, 100 mM NaCl, pH 6) was drop coated onto the ITO electrode and left to equilibrate for 30 min at room temperature. Excess protein was removed by rinsing the electrode with 50 mM MES, 100 mM NaCl, pH 6. Cyclic voltammetry in 50 mM HEPES, 100 mM NaCl, pH 7 was carried out with an Autolab PGSTAT30 instrument controlled by NOVA 2.1.4 software.

### Measurement of FMN Reduction Rates

In brief, 10 mL aliquots of M72 medium supplemented with 20 mM sodium lactate and 30 mM sodium fumarate were inoculated with MR-1 Δ*mtrC/omcA*,^49^ and 600 nM MtrC protein (wtMtrC, MtrC_344BocK_ or MtrC_430BocK_) was added as required. Cultures were grown microaerobically at 30 °C while being shaken (180 rpm). After approximately 15 h growth (OD_600 nm_ approximately 2), the cultures were taken into an N_2_-filled chamber (atmospheric O_2_ < 2 ppm) and transferred to centrifuge tubes. The tubes were sealed and removed from the anaerobic chamber, and the cells were pelleted by centrifugation at 2600*g* for 10 min. Tubes were returned to the anaerobic chamber, where the supernatant was discarded, and the cell pellet was resuspended to OD_600 nm_ ≈ 1.0 in anaerobic *Shewanella* Basal Medium (SBM) supplemented with vitamins and minerals.^43,44^ The resuspended cells were subjected to a further round of centrifugation and anaerobic resuspension, as above, to remove any loosely bound MtrC.

FMN reduction was performed as previously described^43,44^ at room temperature in sealed anaerobic fluorescence cuvettes containing 3 mL of SBM supplemented with vitamins and minerals, 20 mM lactate, and cells at OD_600 nm_ of approximately 0.1. Fluorescence (excitation, 365 nm; emission, 525 nm) was measured over time following addition of FMN to a final concentration of 12 μM. Anaerobic FMN stock solution (1 mM) was prepared in filtered deionized water.

### Click Chemistry Reactions

Copper(I) catalyzed azide–alkyne cycloaddition (CuAAC) reactions were carried out with MtrC_344AlkK_ or MtrC_430AzF_ and azide or alkyne functionalized Cy5 dye, respectively. Protein (10 μM) was incubated with 10× excess of the appropriate Cy5 dye in the presence of 0.1 mM CuSO_4_, 0.5 mM tris(3-hydroxypropyl triazolyl methyl) amine, and 0.5 mM sodium ascorbate in PBS (137 mM NaCl, 2.7 mM KCl, 10 mM Na_2_HPO_4_, 1.8 mM KH_2_PO_4_) pH 7.4. Strain-promoted azide–alkyne cycloaddition (SPAAC) reactions were performed on MtrC_344AzF_ with dibenzocyclooctyne functionalized Cy5 dye in PBS at pH 7.4. Reactions were performed at room temperature. Samples taken for analysis at the desired times were treated with a 4-fold excess of −20 °C acetone and incubated at −20 °C for 20 min to halt the reaction and precipitate the protein to allow its separation from excess reagents. Precipitated protein was pelleted by centrifugation at 12,000*g* for 15 min. The pellet was washed in acetone, and centrifugation was repeated. The pelleted protein was resuspended in SDS-PAGE loading buffer prior to analysis by SDS-PAGE.

## Data Availability

The BocK-MtrC structures and the associated structure factors are deposited in the Protein Data Bank under the access codes 8QC9 for BocK at 239, 8QBZ for BocK at 344, and 8QBQ for BocK at 430. Data sets used to make figures are deposited at Figshare (DOI: 10.6084/m9.figshare.25491769). For the purpose of open access, the authors have applied a CC BY public copyright license to any author-accepted manuscript version that arises.

## Supporting Information Available

Tables of key proteins in the maturation and secretion of MtrC, strains, plasmids, and primers. Plasmid maps, SDS-PAGE gel images, deconvoluted mass spectra, and images of protein structures. Data collection and refinement statistics for crystallographic analysis of MtrC BocK Proteins. Optical densities of selected strains (PDF)
