# Supplementary material for: Genetic Code Expansion in Shewanella oneidensis MR-1 Allows Site-Specific Incorporation of Bioorthogonal Functional Groups into a c-Type Cytochrome
Source: ACS Synth Biol. 2024 Aug 19;13(9):2833–43. doi: 10.1021/acssynbio.4c00248 (PMC11421213; doi:10.1021/acssynbio.4c00248)
Supplement: Supplementary file 1 — sb4c00248_si_001.pdf [file sb4c00248_si_001.pdf]

Supplementary Information

Genetic Code Expansion in *Shewanella oneidensis* MR-1 allows Site-Specific Incorporation of Bioorthogonal Functional Groups into a *c*-type Cytochrome

Colin W. J. Lockwood[1], Benjamin W. Nash[1], Simone E. Newton-Payne[1], Jessica H. van Wonderen[1], Keir P. S. Whiting [1], Abigail Connolly [1], Alexander L. Sutton-Cook[1], Archie Crook[1], Advait R. Aithal[1], Marcus J. Edwards[2], Thomas A. Clarke[1], Amit Sachdeva[1*], Julea N. Butt[1*]

[1] School of Chemistry and School of Biological Sciences, University of East Anglia, Norwich Research Park, Norwich, NR4 7TJ, U.K.

[2] School of Life Sciences, University of Essex, Colchester, CO4 3SQ, U.K.

*to whom correspondence should be addressed: j.butt@uea.ac.uk, a.sachdeva@uea.ac.uk

Tables

Table S1: Key proteins in the maturation and secretion of MtrC with their respective stop codon.

Table S2: Strains and plasmids used in this work.

Table S3: Primers used to introduce the Amber stop codon into *mtrC*.

Table S4: Data collection and refinement statistics for crystallographic analysis of MtrC BocK proteins.

Figures

Figure S1: Plasmid maps.

Figure S2: SDS-PAGE gel images for spent media from culture of MR-1.C, MR-1.Pyl.C and MR-1.*Mj*.C with arabinose (Arb), BocK and AzF.

Figure S3: SDS-PAGE gel images for spent media from culture of MR-1.C, MR-1.Pyl.C and MR-1.*Mj*.C with arabinose (Arb), BocK and AzF.

Figure S4: Deconvoluted mass spectra for proteins purified by affinity chromatography from culture of MR-1.*Mj*.C_{xxxUAG} strains with arabinose and no ncAA.

Figure S5: Crystallographic analysis of BocK containing MtrC proteins.

Figure S6: SDS-PAGE gel images for samples of ncAA-containing MtrC proteins incubated with functionalized Cy5 dyes as indicated.

Table S1. Key proteins in the maturation and secretion of MtrC with their respective stop codon. Amber stop codons (UAG) are highlighted in bold.

Gene	Locus	Product	Stop Codon
		**Heme biosynthesis pathway**	
hemG	SO_0027	oxygen-independent protoporphyrinogen oxidase HemG	UAA
hemF	SO_0038	aerobic coproporphyrinogen III oxidase HemF	**UAG**
hemL	SO_1300	glutamate-1-semialdehyde-21-aminomutase HemL	UAA
hemH-1	SO_2019	ferrochelatase HemH	UGA
hemB-1	SO_2587	delta-aminolevulinic acid dehydratase HemB	UAA
hemK	SO_3080	modification methylase, HemK family hemK	UAA
hemH-2	SO_3348	ferrochelatase HemH	UAA
hemA	SO_3834	glutamyl-tRNA reductase HemA	UAA
hemB-2	SO_4208	delta-aminolevulinic acid dehydratase HemB	UAA
hemC	SO_4313	hydroxymethylbilane synthase HemC	UAA
hemD	SO_4314	uroporphyrinogen-III synthase HemD	UAA
hemX	SO_4315	uroporphyrin-III C-methyltransferase HemX	UGA
hemE	SO_0435	uroporphyrinogen decarboxylase HemE	UAA
hemN	SO_4730	coproporphyrinogen III oxidase oxygen-independent HemN	UAA
		**Cytochrome c Maturation**	
ccmE	SO_0259	ABC-type heme export system chaperone component CcmE	UAA
ccmD	SO_0260	ABC-type heme export system CcmE-interacting component CcmD	UGA
ccmC	SO_0261	ABC-type heme export system permease component 2 CcmC	UAA
ccmB	SO_0262	ABC-type heme export system permease component 1 CcmB	UAA
ccmA	SO_0263	ABC-type heme export system ATPase component CcmA	UAA
ccmI	SO_0265	apo-cytochrome c chaperone CcmI	UAA
ccmF	SO_0266	cytochrome c synthetase cytochrome b containing quinol-haem oxidoreductase subunit CcmF	UAA
ccmG	SO_0267	cytochrome c maturation system membrane anchored thioredoxin CcmG	UGA
ccmH	SO_0268	cytochrome c synthetase subunit CcmH	UGA
		**Sec system**	
secB	SO_0052	protein export chaperone SecB	UAA
secE	SO_0218	preprotein translocase subunit SecE	**UAG**
secY	SO_0251	preprotein translocase subunit SecY	UAA
secD-1	SO_1193	preprotein translocase subunit SecD-1	UAA
secF-1	SO_1194	preprotein translocase subunit SecF-1	**UAG**
secG	SO_1201	preprotein translocase subunit SecG	UAA
secF-2	SO_3110	preprotein translocase subunit SecF-2	UAA
secD-2	SO_3111	preprotein translocase subunit SecD-2	UAA
yajC	SO_3112	SecDF preprotein translocase-associated protein YajC	UAA
secA	SO_4211	preprotein translocase ATPase subunit SecA	UAA

continues on next page

Table S1 *continued*.

Gene	Locus	Product	Stop Codon
		**Type 2 secretion system**	
gspC	SO_0165	T2aSS secretion system protein GspC	UAA
gspD	SO_0166	T2aSS secretion system secretin GspD	UGA
gspE	SO_0167	T2aSS secretion system assembly ATPase GspE	UAA
gspF	SO_0168	T2aSS secretion system inner membrane platform protein GspF	**UAG**
gspG	SO_0169	T2aSS secretion system pseudopilus protein GspG	UAA
gspH	SO_0170	T2aSS secretion system pseudopilus protein GspH	**UAG**
gspI	SO_0171	T2aSS secretion system pseudopilus protein GspI	UAA
gspJ	SO_0172	T2aSS secretion system protein GspJ	UGA
gspK	SO_0173	T2aSS secretion system pseudopilus protein GspK	UAA
gspL	SO_0174	T2aSSsecretion system inner membrane platform protein GspL	UAA
gspM	SO_0175	T2aSS secretion system inner membrane platform protein GspM	**UAG**
gspN	SO_0176	T2aSS secretion system protein GspN	UAA
		**Metal reducing pathway**	
mtrB	SO_1776	extracellular iron oxide respiratory system outer membrane MtrB	UAA
mtrA	SO_1777	extracellular iron oxide respiratory system periplasmic decaheme cytochrome c component MtrA	UAA
mtrC	SO_1778	extracellular iron oxide respiratory system surface decaheme cytochrome c component MtrC	UAA
omcA	SO_1779	extracellular iron oxide respiratory system surface decaheme cytochrome c component OmcA	UAA
mtrF	SO_1780	extracellular respiratory system surface decaheme cytochrome c component MtrF	UAA
mtrE	SO_1781	extracellular respiratory system outer membrane component MtrE	UAA
mtrD	SO_1782	extracellular respiratory system periplasmic decaheme cytochrome c component MtrD	UAA

Table S2. Strains and plasmids used in this work.

Strains	Relevant feature	Source/Reference
S. oneidensis		
MR-1	*Wildtype strain*	Lab stock
LS527	Δ*mtrB-mtrD,* locus tags SO_1776-SO_1782	Lab stock
LS789	Δ*mtrC-omcA,* locus tags SO_1778-SO_1779	Lab stock
MR-1.C	MR-1 containing pBAD.C<br>(pBAD.C previously termed pJvW001)	*(1)*
MR-1.Pyl.C	MR-1 containing pBAD.Pyl.C	This work
MR-1.Pyl.C_{293UAG}	MR-1 containing pBAD.Pyl.C_{293UAG}	This work
MR-1.Pyl.C_{344UAG}	MR-1 containing pBAD.Pyl.C_{344UAG}	This work
MR-1.Pyl.C_{430UAG}	MR-1 containing pBAD.Pyl.C_{430UAG}	This work
MR-1.*Mj*.C	MR-1 containing pBAD.*Mj*.C	This work
MR-1.*Mj*.C_{293UAG}	MR-1 containing pBAD.*Mj*.C_{293UAG}	This work
MR-1.*Mj*.C_{344UAG}	MR-1 containing pBAD.*Mj*.C_{344UAG}	This work
MR-1.*Mj*.C_{430UAG}	MR-1 containing pBAD.*Mj*.C_{430UAG}	This work
E. coli		
Top10	Cloning strain	Lab stock

Plasmids	Relevant feature	Source/Reference
pBAD.C	pBAD/TOPO derivative encoding WT $MtrC^{Cstrp}$<br>Previously referred to as pJvW001	*(1)*
pBAD.Pyl.C	pBAD/TOPO derivative encoding<br>*Mb*PylRS/PylT and WT $MtrC^{Cstrp}$	This work
pBAD.Pyl.C_{293UAG}	pBAD/TOPO derivative encoding<br>*Mb*PylRS/PylT and A293UAG (STOP) $MtrC^{Cstrp}$	This work
pBAD.Pyl.C_{344UAG}	pBAD/TOPO derivative encoding<br>*Mb*PylRS/PylT and E344UAG (STOP) $MtrC^{Cstrp}$	This work
pBAD.Pyl.C_{430UAG}	pBAD/TOPO derivative encoding<br>*Mb*PylRS/PylT and A430UAG (STOP) $MtrC^{Cstrp}$	This work
pBAD.*Mj*.C	pBAD/TOPO derivative encoding<br>*Mj*CNFRS/tRNA and WT $MtrC^{Cstrp}$	This work
pBAD.*Mj*.C_{293UAG}	pBAD/TOPO derivative encoding *Mj*CNFRS<br>/tRNA and A293UAG (STOP) $MtrC^{Cstrp}$	This work
pBAD.*Mj*.C_{344UAG}	pBAD/TOPO derivative encoding *Mj*CNFRS<br>/tRNA and E344UAG (STOP) $MtrC^{Cstrp}$	This work
pBAD.*Mj*.C_{430UAG}	pBAD/TOPO derivative encoding *Mj*CNFRS<br>/tRNA and A430UAG (STOP) $MtrC^{Cstrp}$	This work
pAS61	*Mb*PylRS/PylT	*(2)*
pAS76	*Mj*CNFRS /tRNA	*(2)*

Table S3. Primers used to introduce the Amber stop codon into *mtrC*.

Primer	Sequence (5'→3')	Description
AS-RS/tRNA Forward	GCATCTGTGCGGTATTTCACACCGCAGGATCCTCGGGAGTTGTCAG	Primers to amplify PylRS/tRNA from pAS61 and *Mj*CNFRS/tRNA from pAS76. PCR products used in Gibson cloning.
AS-RS/tRNA Reverse	GCAGATTGTACTGAGAGTGCACCATAGTTGGGTAACGCCAGGGTTTTC	
$MtrC_{A293TAG}$ Forward	GACATCGATTTTGCT**TAG**GGTAAAGGC	Introduced amber stop codon at position A293 in pJvW001
$MtrC_{A293TAG}$ Reverse	GCCTTTACC**CTA**AGCAAAATCGATGTC	
$MtrC_{E344TAG}$ Forward	CAATTAATACC**TAG**ACTAAAGCAG	Introduced amber stop codon at position E344 in pJvW001
$MtrC_{E344TAG}$ Reverse	CTGCTTTAGT**CTA**GGTATTAATTG	
$MtrC_{A430TAG}$ Forward	AAAACGGC**TAG**GACAGCGA	Introduced amber stop codon at position E344 in pJvW001
$MtrC_{A430TAG}$ Reverse	TCGCTGTC**CTA**GCCGTTTT	

Table S4. Data collection and refinement statistics for crystallographic analysis of MtrC BocK Proteins (with the corresponding PDB accession code).

	MtrC-293 BocK (8QC9)	MtrC-344 BocK (8QBZ)	MtrC-430 BocK (8QBQ)
**Data collection**			
Space group	P 2_1 2_1 2_1	P 2_1 2_1 2_1	P 2_1 2_1 2_1
Cell dimensions			
a, *b*, *c* (Å)	53.08, 90.02, 154.52	52.97, 89.66, 153.55	52.90, 89.61, 154.03
α, β, γ (°)	90.00, 90.00, 90.00	90.00, 90.00, 90.00	90.00, 90.00, 90.00
Resolution (Å)	58.66-2.00 (2.00-2.05)	89.66-1.90 (1.90-1.93)	58.41-1.81 (1.84 - 1.81)
$CC_{1/2}$ (%)	99.1 (60.2)	92.0 (48.3)	99.8 (89.3)
$I / \sigma I$	6.1 (1.4)	5.3 (1.2)	14.0 (2.0)
Completeness (%)	100 (100)	99.7 (92.3)	100 (97.72)
Multiplicity	13.4 (13.9)	11.3 (10.7)	13.0 (9.5)
**Refinement**			
Resolution (Å)	2.00	1.90	1.81
No. reflections	50847	58497	67485
R_{work} / R_{free}	0.173/0.214	0.174/0.216	0.155/0.187
No. atoms			
Protein	4719	4732	4722
Ligand/ion	467	482	491
Water	785	959	978
B-factors			
Protein	24.47	18.42	20.36
Ligand/ion	20.50	15.78	17.69
Water	31.44	30.76	30.26
R.m.s. deviations			
Bond lengths (Å)	0.009	0.021	0.008
Bond angles (°)	1.10	2.18	1.06

*Values in parentheses are for highest-resolution shell.

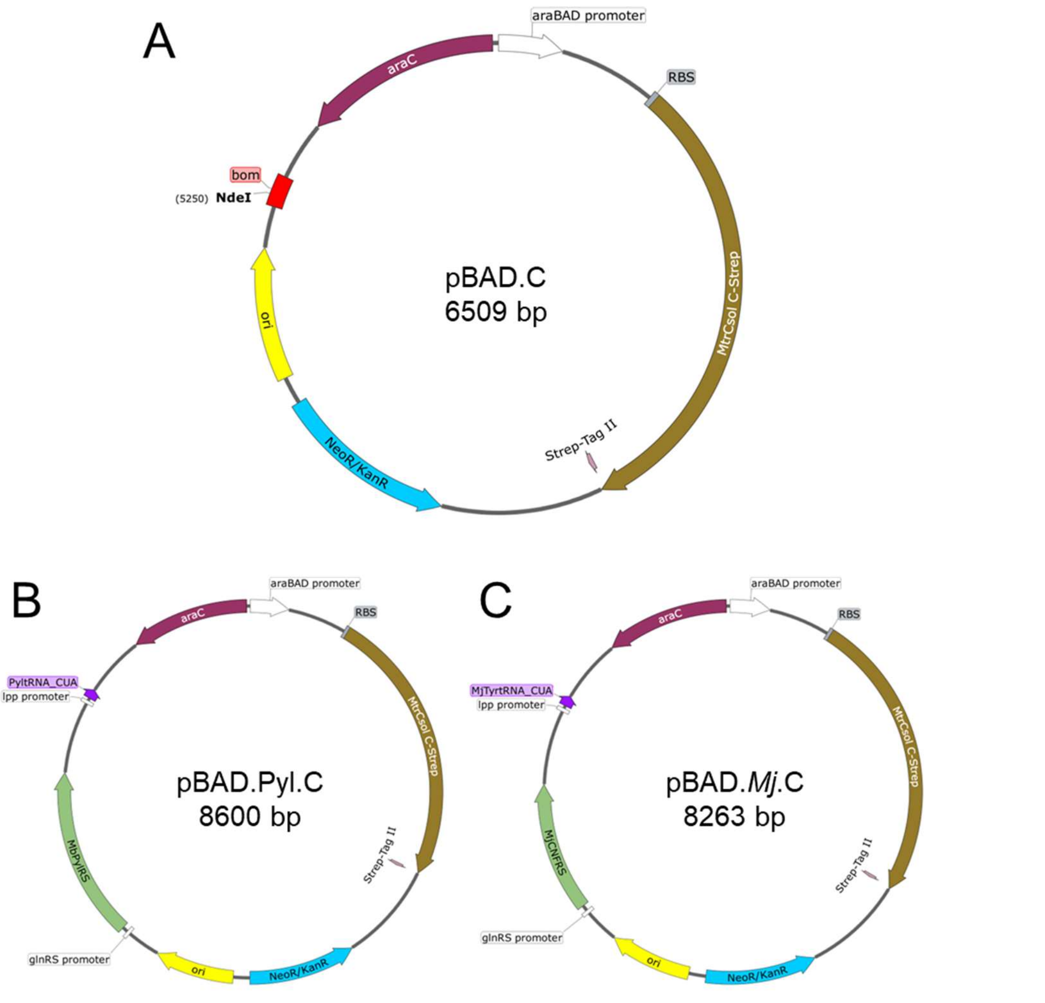

Figure S1. Plasmid maps.

(A) pBAD.C. The highlighted Nde1 site within the basis of motility site (BOM) was used for insertion of a DNA fragment containing the RS/tRNA.

(B) pBAD.Pyl.C. Modified pBAD.C plasmid containing the *Methanosarcina barkeri* pyrrolysyl-tRNA synthetase/tRNA$_{Pyl}^{CUA}$ (*Mb*PylRS/tRNA^{CUA}) pair, under the GlnRS and lpp promoters, respectively.

(C) pBAD.*Mj*.C. pBAD.C plasmid incorporating the *Methanocaldococcus jannaschii* tyrosyl-tRNA synthetase/tRNA^{CUA} (*Mj*CNFRS/tRNA^{CUA}) under the GlnRS and lpp promoters, respectively.

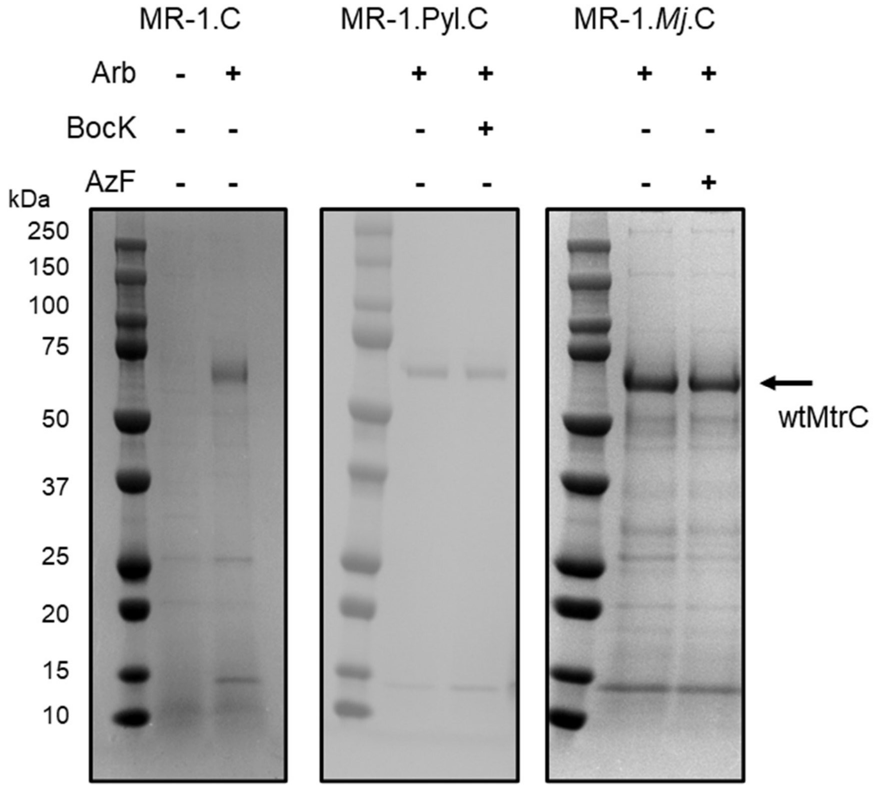

Figure S2. SDS-PAGE gel images for spent media from culture of MR-1.C, MR-1.Pyl.C and MR-1.*Mj*.C with arabinose (Arb), BocK and AzF as indicated. Proteins visualized by Coomassie stain. Samples correspond to those of Figure 3 in the main text. Arrow indicates the expected migration of wtMtrC.

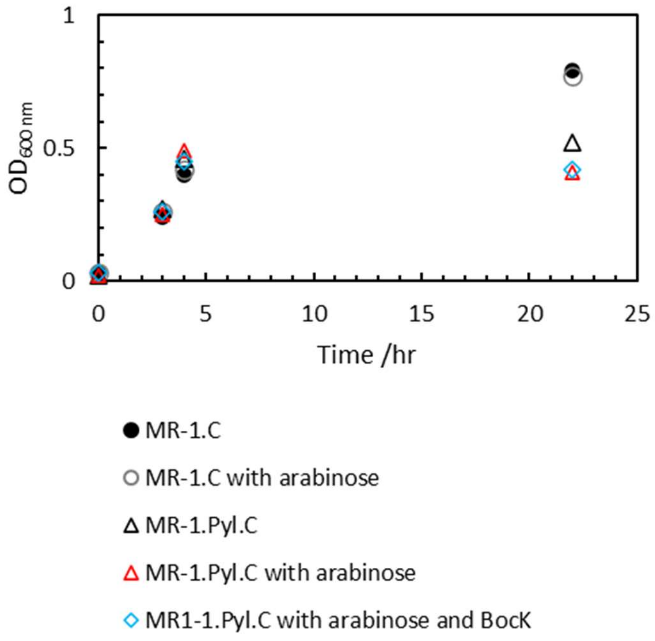

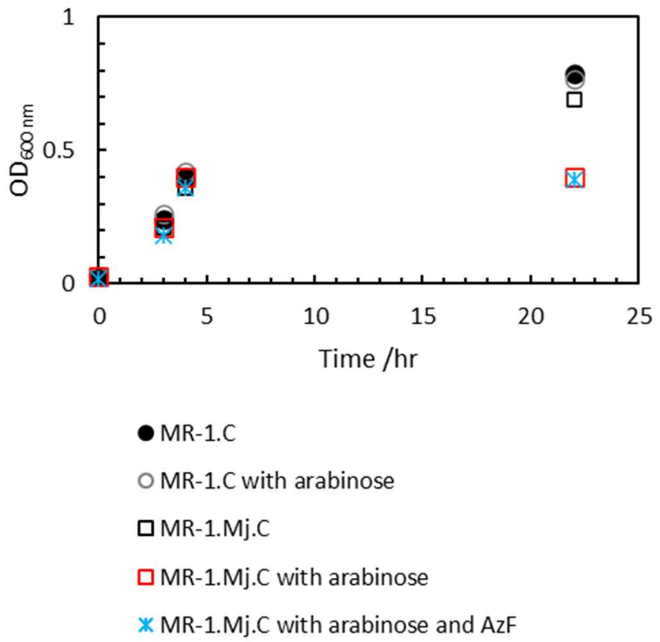

Figure S3. Optical density (OD) at 600 nm for the indicated cultures. At $OD_{600\ nm}$ approximately 0.4, arabinose was added to a final concentration of 5 mM and the indicated ncAA was added to a final concentration of 4 mM.

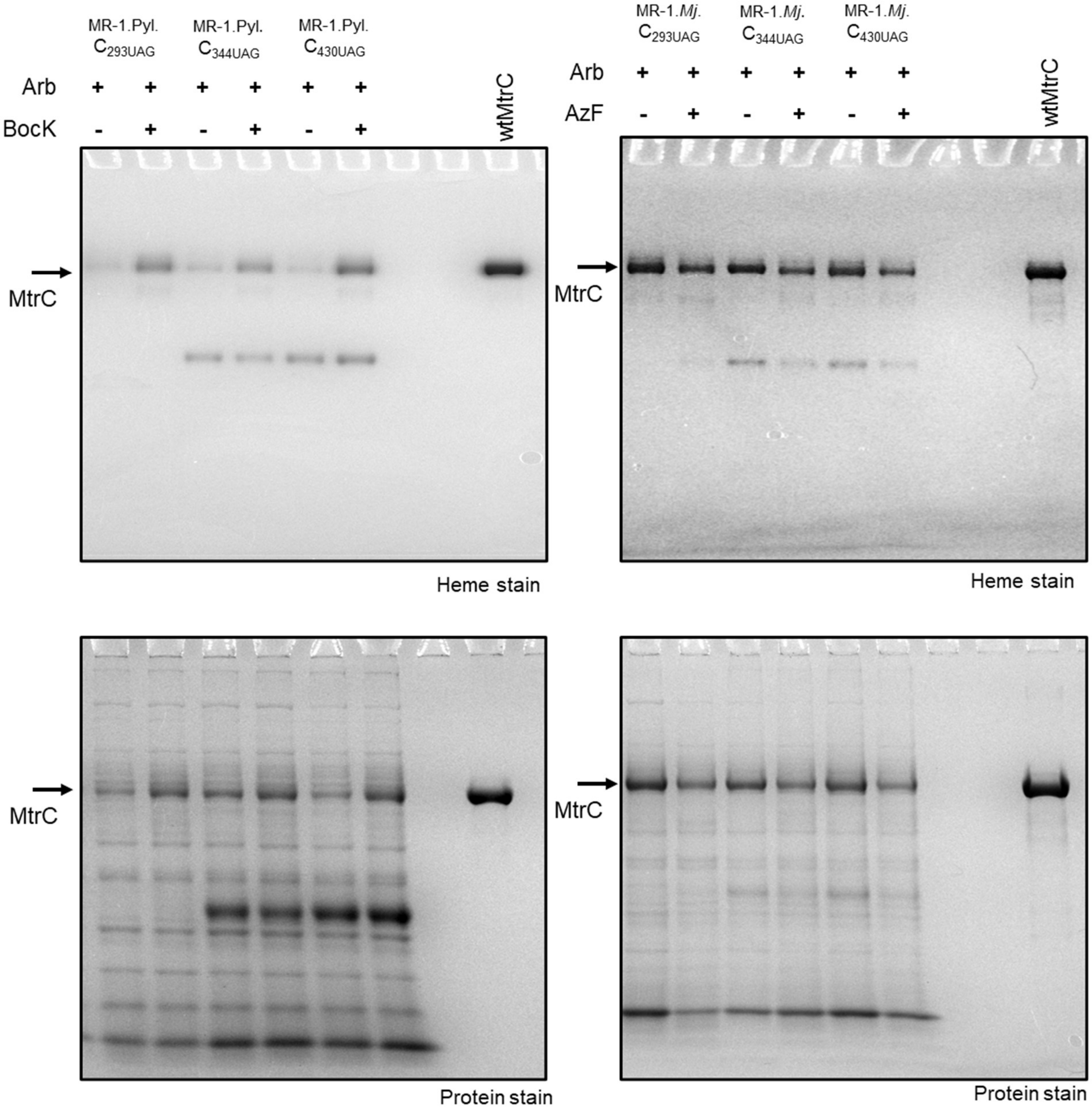

Figure S4. SDS-PAGE gel images for spent media from culture of MR-1.C, MR-1.Pyl.C and MR-1.*Mj*.C with arabinose (Arb), BocK and AzF as indicated. Proteins visualized with heme stain (upper) and Coomassie stain (lower). Samples correspond to those presented in Figure 4 of the main text. Arrows indicate the migration of wtMtrC.

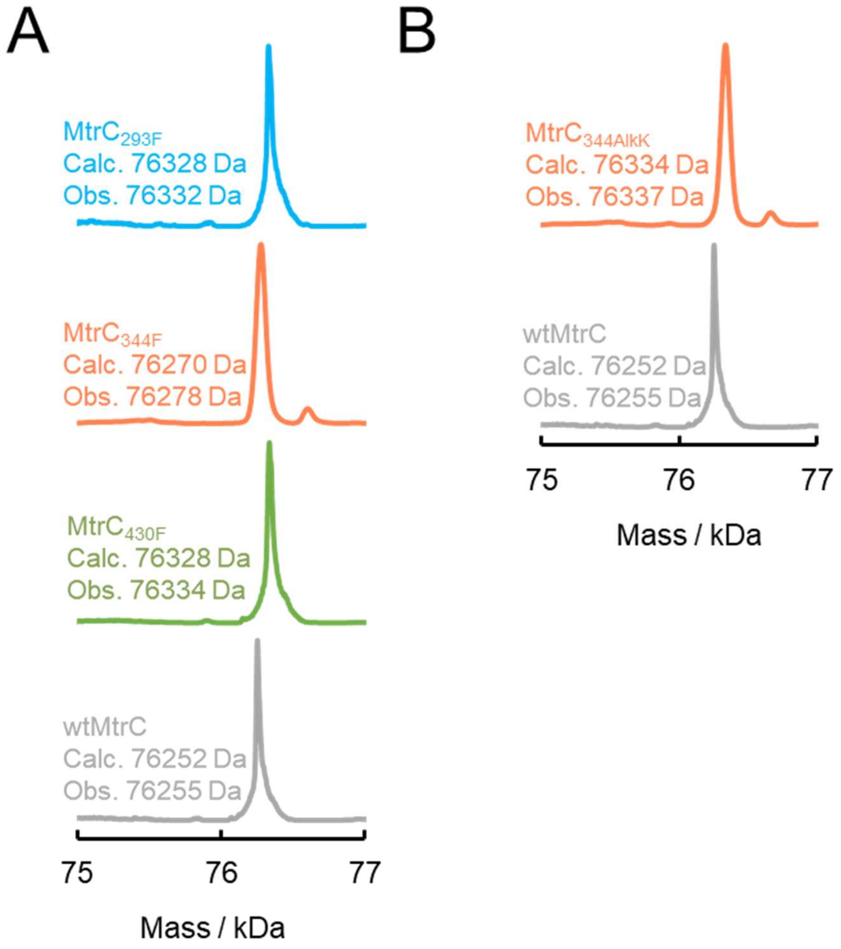

Figure S5. Deconvoluted mass spectra for MtrC proteins. A) Proteins purified by affinity chromatography from culture of MR-1.*Mj*.C_{xxxUAG} strains with arabinose and no ncAA. Intact mass values are consistent with insertion of phenylalanine at the site encoded by the amber stop codon: calculated (calc.) and observed (obs.). Thus, spectra are labelled for the corresponding protein $MtrC_{xxxF}$ where xxx is the residue encoded by the amber stop codon. The deconvoluted mass spectrum of wtMtrC is included for reference. **B)** $MtrC_{344AlkK}$ protein purified by affinity chromatography from a culture of MR-1.pyl.$_{344xUAG}$ with arabinose and alkyne lysine. The deconvoluted mass spectrum of wtMtrC is included for reference.

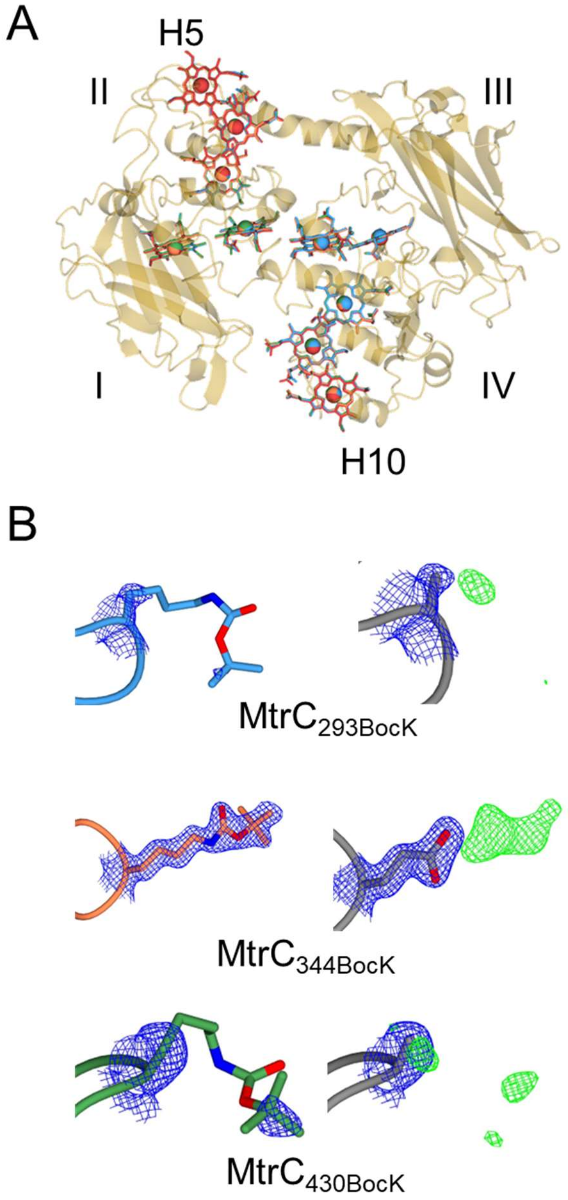

Figure S6. Crystallographic analysis of BocK containing MtrC proteins.

(A) Pairwise alignment of the heme cofactors in $MtrC_{293BocK}$ (Blue), $MtrC_{344BocK}$ (coral) and $Mtr_{430BocK}$ (green) and wtMtrC (red) overlaid on the secondary structure of wtMtrC (gold). H5 indicates Heme 5 and H10 indicates Heme 10 where Hemes are numbered in order of their sites of attachment to the MtrC peptide, protein domains are labelled I- IV.

(B) The 2Fo-Fc (blue) and Fo-Fc (green/red) electron density map (contoured to 1.2 and 3.5 sigma respectively) for the indicated proteins, resulting from (left) refinement of the BocK MtrC structure model against the BocK MtrC data and (right) refinement of the wtMtrC structure (PDB ID: 4LM8) against the BocK MtrC data.

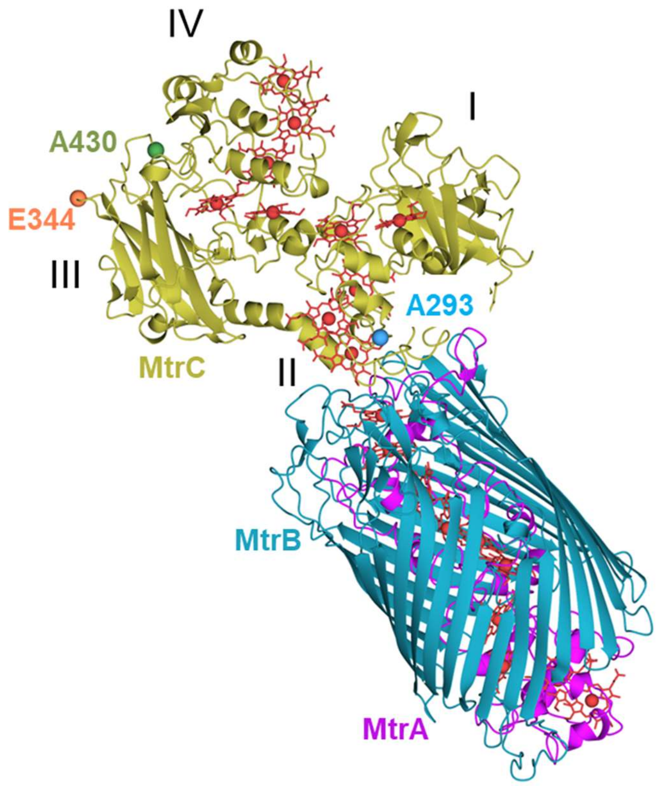

Figure S7. Alpha-fold model of *S. oneidensis* MR-1 MtrCAB. Outer membrane spanning MtrA (purple) and MtrB (blue) with extracellular MtrC (gold). Domains I to IV of MtrC are indicated, hemes are red and the C_α carbons of residues 293, 344 and 430 are shown as blue, salmon and green spheres, respectively.

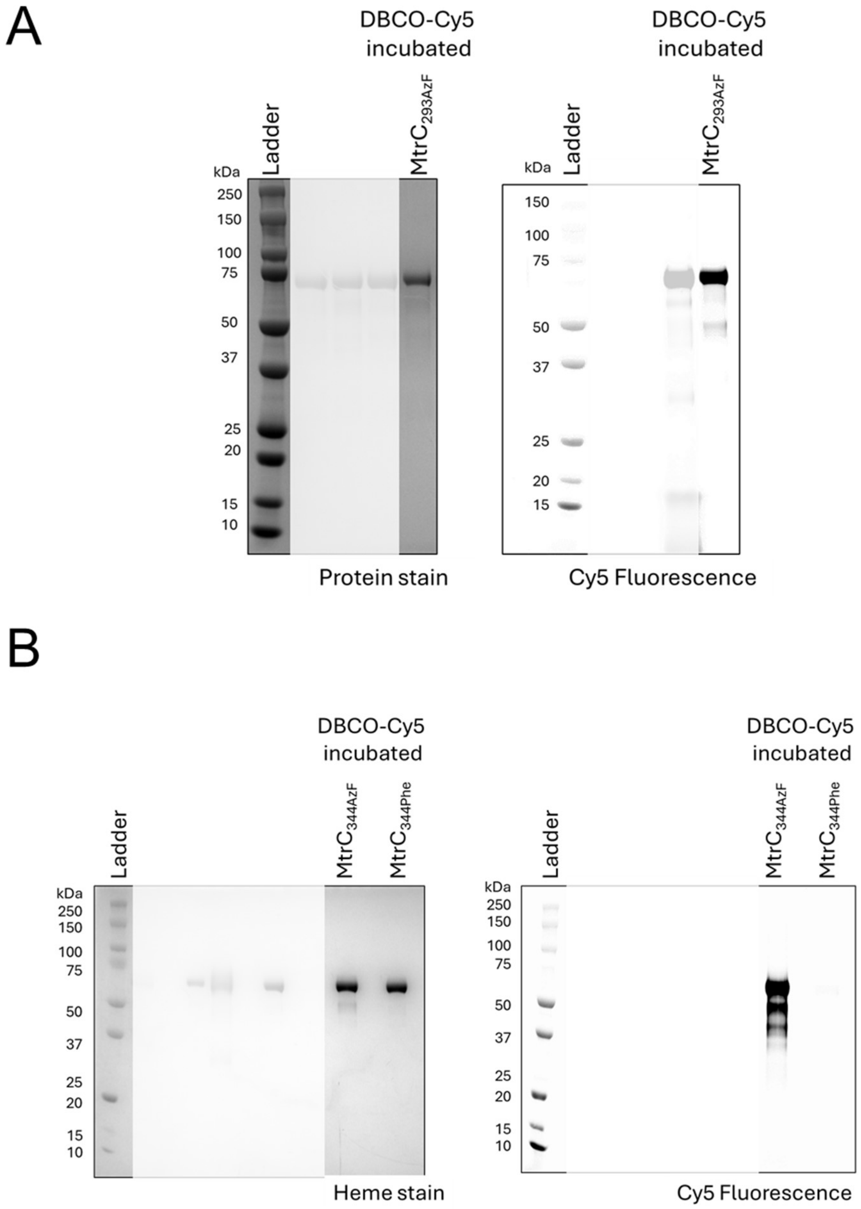

Figure S8. Introduction of fluorescent probes to AzF-containing MtrC proteins using bioorthogonal chemistry. SDS-PAGE gel images for the reaction products from incubation of dibenzocyclooctyne sulfo-cyanine 5 (DBCO-Cy5) with (A) $MtrC_{293AzF}$ and (B) $MtrC_{344AzF}$ and $MtrC_{344Phe.}$ Gels imaged by protein stain, heme stain or fluorescence emission (excitation at 635 nm) as indicated. Masked lanes carry samples not relevant to this study. Reaction time = 18 hours.

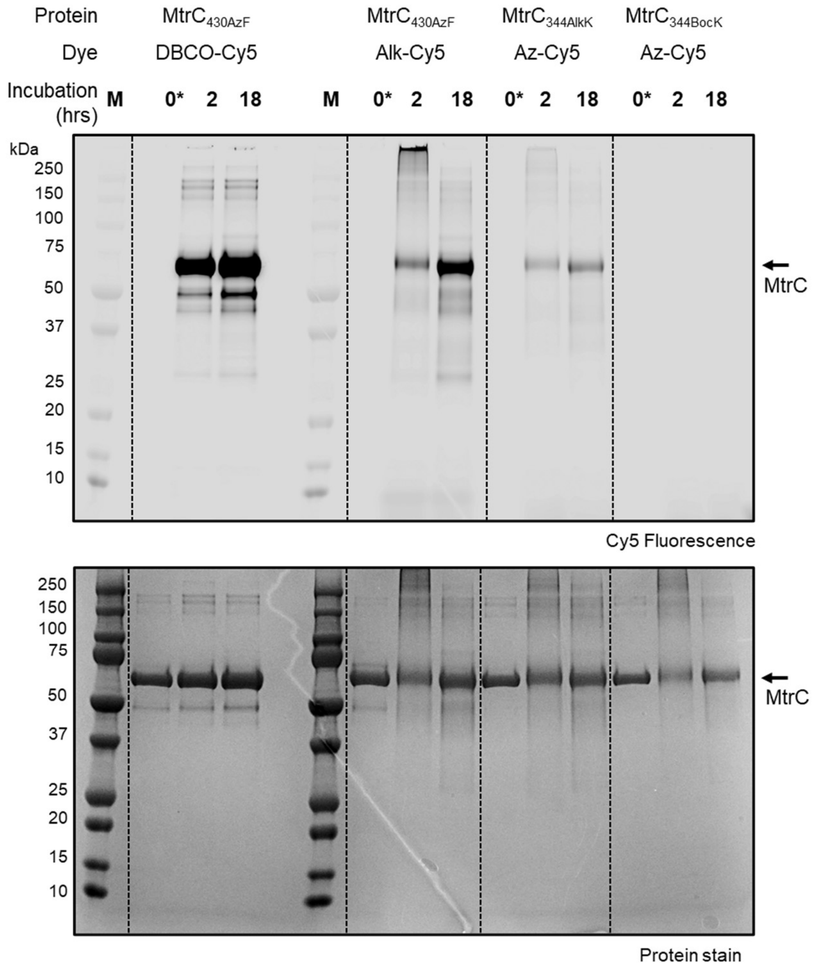

Figure S9. SDS-PAGE gel images for samples of ncAA-containing MtrC proteins incubated with functionalized Cy5 dyes as indicated. Top: Cy5 dye visualized by fluorescence emission (excitation at 635 nm). Bottom: proteins visualized by Coomassie stain. Arrows indicate the expected migration of MtrC proteins.

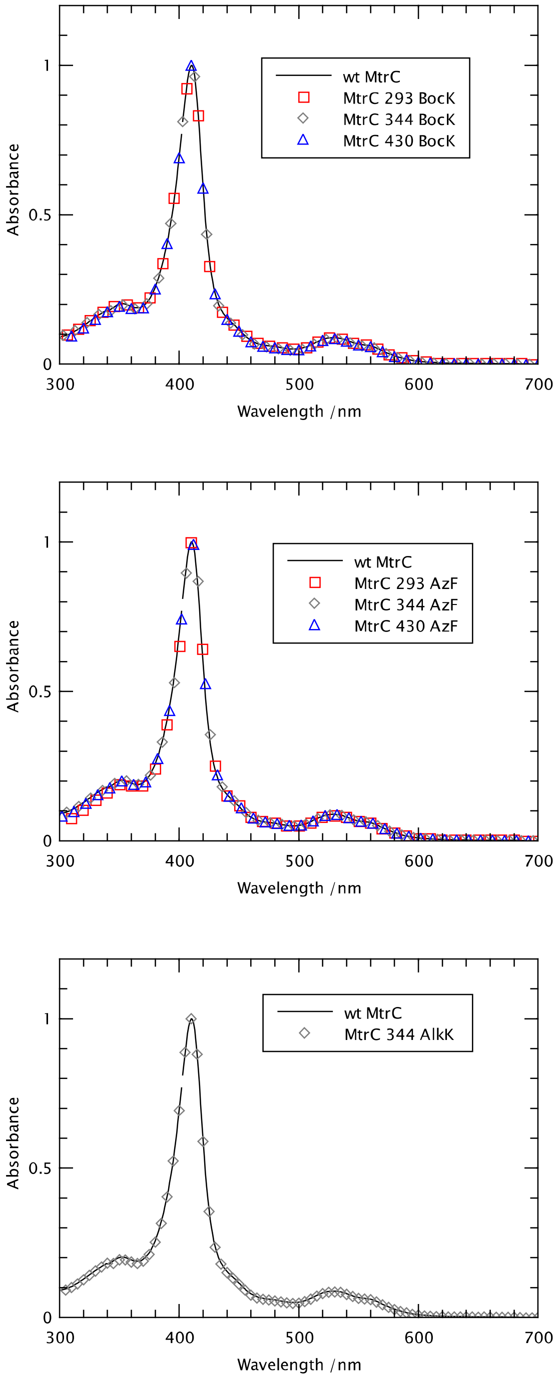

Figure S10. Spectroscopic analysis of ncAA containing MtrC proteins. The electronic absorbance spectrum of oxidized (air equilibrated) wtMtrC compared to that of the BocK, AzF and Alk containing proteins as indicated. Samples MtrC (0.5 to 0.8 µM) in 100 mM Tris-HCl, 150 mM NaCl, pH 8.5.

References

1. Lockwood, C. W. J., van Wonderen, J. H., Edwards, M. J., Piper, S. E. H., White, G. F., Newton-Payne, S., Richardson, D. J., Clarke, T. A., and Butt, J. N. (2018) Membrane-spanning electron transfer proteins from electrogenic bacteria: production and investigation, *Meth. Enzymol. 613*, 257-275.
2. Bridge, T., Wegmann, U., Crack, J. C., Orman, K., Shaikh, S. A., Farndon, W., Martins, C., Saalbach, G., and Sachdeva, A. (2023) Site-specific encoding of photoactivity and photoreactivity into antibody fragments, *Nat. Chem. Biol. 19*, 740-749.
